# Vinylogous functionalization of 4-alkylidene-5-aminopyrazoles with methyl trifluoropyruvates

**DOI:** 10.3762/bjoc.21.41

**Published:** 2025-03-10

**Authors:** Judit Hostalet-Romero, Laura Carceller-Ferrer, Gonzalo Blay, Amparo Sanz-Marco, José R Pedro, Carlos Vila

**Affiliations:** 1 Departament de Química Orgànica, Facultat de Química, Universitat de València, Dr Moliner 50, 46100 Burjassot, València, Spain

**Keywords:** alcohols, diastereoselectivity, nitrogen heterocycles, pyrazoles, vinylogous reaction

## Abstract

A valuable vinylogous addition reaction between 4-alkylidene-5-aminopyrazoles and alkyl trifluoropyruvates leading to highly functionalized tertiary alcohols bearing a trifluoromethyl group and a pyrazole ring is presented. The corresponding trifluoromethyl alcohols are obtained in moderate to good yields (up to 80%) and high diastereoselectivity (up to 7:1).

## Introduction

**Figure 1:**
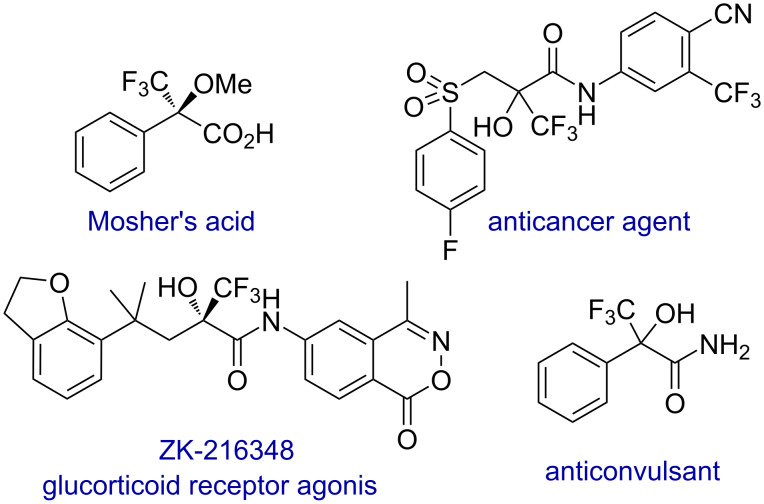
Biologically active compounds featuring a trifluoromethyl carbinol motif.

Vinylogy refers to the transmission of electronic effects through a conjugated π-system, enabling the extension of a functional group's nucleophilic or electrophilic properties along a C=C double bond [1]. This effect has been established to be very advantageous to expand the range of reactions of different functional groups that can be coupled efficiently through a conjugated π-system. In this context, the addition reaction of vinylogous nucleophiles to carbonyl compounds is a significant and important reaction for the selective synthesis of homoallylic alcohols in an efficient and sustainable way [2–3]. As carbonyl compounds, alkyl trifluoropyruvates [4–5] are an interesting class of compounds that have been used in addition reactions of different nucleophiles for the synthesis of tertiary trifluoromethyl carbinols [6–7]. In this context, trifluoromethyl carbinols constitute a key structural motif present in a wide range of molecules with important biological activities (Figure 1) [8–10], on account of the distinctive properties of organofluorine compounds that generally enhance the bioactivity of agrochemical and pharmaceutical substrates.

**Figure 2:**
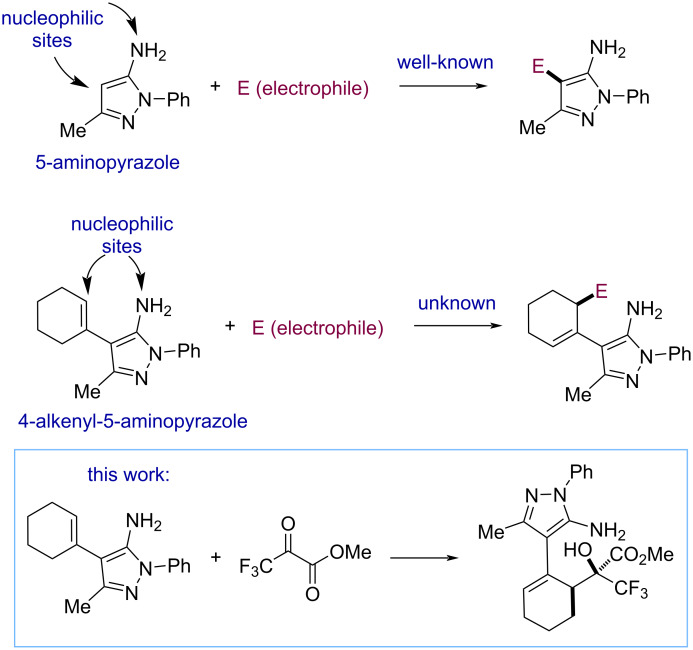
Nucleophilic sites of 5-aminopyrazoles and 4-alkenyl-5-aminopyrazoles. Stereoselective synthesis of trifluoromethyl carbinols through an vinylogous addition reaction of 4-alkenyl-5-aminopyrazoles to alkyl trifluoropyruvates.

On the other hand, 5-aminopyrazole [11–12] is a nitrogen heterocycle that has attracted significant interest to pharmaceutical and medicinal chemists due to the presence of this nitrogen heterocycle in various biologically active compounds, particularly antibacterial and antifungal agents [13–14]. This class of functionalized nitrogen heterocycles is notable for its synthetic versatility, because it shows different nucleophilic positions, making regioselectivity a synthetic challenge. Numerous studies have reported on the regioselective electrophilic functionalization of this nitrogen heterocycle [15–25]. However, the vinylogous functionalization of 5-aminopyrazoles has not been described to the best of our knowledge (Figure 2). As a part of our ongoing interest in the functionalization of 5-aminopyrazoles [26], we decided to study 4-alkenyl-5-aminopyrazoles as nucleophiles in the vinylogous addition reaction to electrophiles. Herein, we report the regioselective and diastereoselective functionalization of 4-cyclohexenyl-5-aminopyrazoles using alkyl trifluoropyruvates [27–29] as electrophiles. It is noteworthy that the development of such vinylogous functionalizations of this nitrogen heterocycle with a fluorine-containing electrophile may be of interest to pharmaceutical and medicinal chemists.

## Results and Discussion

**Scheme 1:**
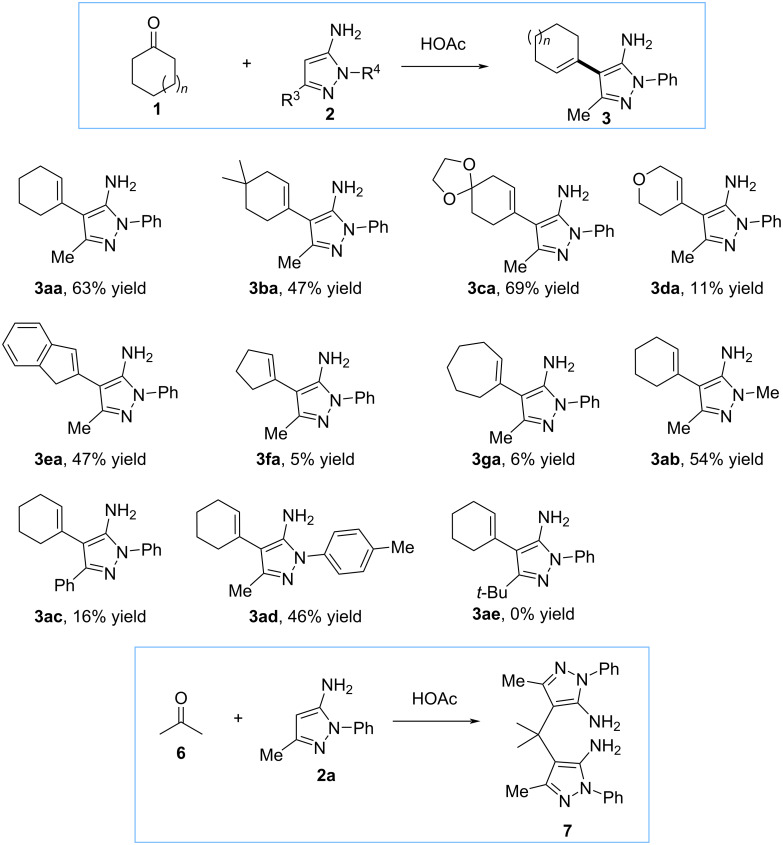
Synthesis of the starting materials **3**.

4-(Alkenyl)-5-aminopyrazoles **3** were selected as starting materials to study the vinylogous functionalization with alkyl trifluoropyruvates. The synthesis of compounds **3** was accomplished by the reaction of cyclic ketones **1** and 5-aminopyrazoles **2** in the presence of acetic acid (Scheme 1) [30–31]. Cyclohexenones **1a**–**c** provided the corresponding products **3aa**–**ca** in good yields (47–69%). On the other hand, the reaction with tetrahydro-4*H*-pyran-4-one (**1d**) occurred with a significant decrease in yield, dropping to 11%. The yield is also affected by the number of carbon atoms of the starting cyclic ketone **1**. In the case of structure **3ea**, which involves 2-indanone, the decrease in yield is not very pronounced. However, when cyclopentanone (**1f**) or cycloheptanone (**1g**) are used, the yields dropped significantly to 5% and 6%, respectively.

Next, maintaining cyclohexanone as a cyclic ketone, a series of compounds **3** were synthesized by modifying the 5-aminopyrazole **2**. Compounds **3ab** and **3ad** were obtained in a comparable yield (54 and 46%) from 1,3-dimethyl-1*H*-pyrazol-5-amine (**2b**) or 3-methyl-1-(*p*-tolyl)-1*H*-pyrazol-5-amine (**2d**). On the other hand, 1,3-diphenyl-1*H*-pyrazol-5-amine (**2c**) provided the corresponding product **3ac** in much lower yield (16%). If a *tert*-butyl group is present in the C-3 position of the pyrazole as in the case of substrate **2e**, the reaction did not take place, likely due to a considerable increase in steric hindrance. We also attempted to synthesize 4-(alkenyl)-5-aminopyrazoles using an acyclic ketone, such as acetone, but unfortunately, the reaction yielded compound **7** as the final product.

Once the starting materials were synthesized, we focused our attention on the optimization of the reaction conditions. We chose the reaction between 4-cyclohexenyl-5-aminopyrazole (**3aa**) and methyl trifluoropyruvate (**4a**) for the optimization studies (Table 1). First, we tried several solvents (dichloromethane, toluene and dichloroethane, entries 1–3 in Table 1) at room temperature, obtaining product **5aaa** in yields around 50% with high diastereoselectivity (up to 6:1) after several days. Increasing the temperature to 50 °C (Table 1, entries 4 and 5), reduced the reaction time obtaining similar yields for compound **5aaa**. When the reaction was performed at 70 °C in toluene (Table 1, entry 6), after 24 hours, a full conversion of compound **3aa** was observed, affording the corresponding alcohol **5aaa** in 66% yield and 7:1 dr. Other solvents such as dichloroethane, chloroform or ethyl acetate gave lower yields but similar diastereoselectivity. More polar solvents such THF and CH_3_CN afforded the corresponding alcohol **5aaa** with lower diastereoselectivity. When acetone was used as solvent, product **5aaa** was not detected in the ^1^H NMR of crude reaction mixture [32]. Then, we increased the reaction scale to 0.2 mmol and obtained similar results (Table 1, entry 13). At this point, we decided to explore the use of a bifunctional organocatalyst in order to improve the yield. When squaramide **SQ-1** was used as a catalyst, we observed a similar yield and diastereoselectivity after 24 h of reaction (61% yield and 7:1 dr, Table 1, entry 14). By lowering the reaction temperature to 50 °C using the same catalyst (Table 1, entry 15), the yield of the reaction increased slightly to 73% in 24 hours. Disappointingly, the bifunctional thiourea **THIO-1** gave a lower yield and diastereoselectivity at 50 °C (Table 1, entry 16). Finally, the addition of molecular sieves was evaluated (Table 1, entries 17 and 18) affording in both cases lower yields for the reaction product. We also attempted asymmetric reactions using chiral organocatalysts to achieve an enantioselective outcome; however, we observed only racemic mixtures, likely due to the occurrence of a background reaction (details for asymmetric trials are provided in Supporting Information File 1). On the view of the results of the optimization process, we decided to study the reaction scope using the reaction conditions of entries 10 and 12 in Table 1.

**Table 1:** Optimization of the reaction conditions.^a^

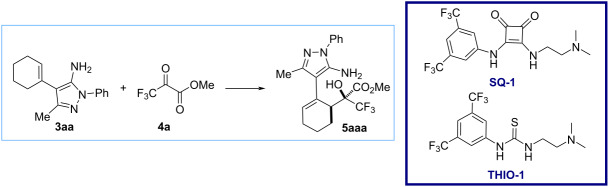						

Entry	Solvent	Cat. (5 mol %)	*T* (°C)	*t* (h)	Yield **3aa** (%)^b^	dr^c^

1	CH_2_Cl_2_	–	rt	96	52	5:1
2	toluene	–	rt	120	48	6:1
3	ClCH_2_CH_2_Cl	–	rt	96	51	6:1
4	toluene	–	50	72	50	5:1
5	ClCH_2_CH_2_Cl	–	50	48	55	5:1
6	toluene	–	70	24	66	7:1
7	ClCH_2_CH_2_Cl	–	70	24	59	7:1
8	CHCl_3_	–	70	24	46	6:1
9	EtOAc	–	70	24	26	6:1
10	THF	–	70	24	65	3.5:1
11	acetone	–	70	24	n. d.	–
12	CH_3_CN	–	70	24	58	3:1
13^d^	toluene	–	70	24	64	7:1
14^d^	toluene	**SQ-1**	70	24	61	7:1
15^d^	toluene	**SQ-1**	50	24	73	7:1
16^d^	toluene	**THIO-1**	50	24	55	4:1
17^d,e^	toluene	–	70	24	44	7:1
18^d,e^	toluene	**SQ-1**	50	24	61	7:1

^a^Reaction conditions: **3aa** (0.1 mmol) and **4a** (0.3 mmol) in 1 mL of solvent at *T* (°C); ^b^isolated yield after column chromatography; ^c^determined by ^1^H NMR of the crude reaction mixture; ^d^reaction conditions: **3aa** (0.2 mmol) and **4a** (0.6 mmol) in 1 mL of solvent at *T* (°C); ^e^50 mg of molecular sieves 4 Å were used.

**Scheme 2:**
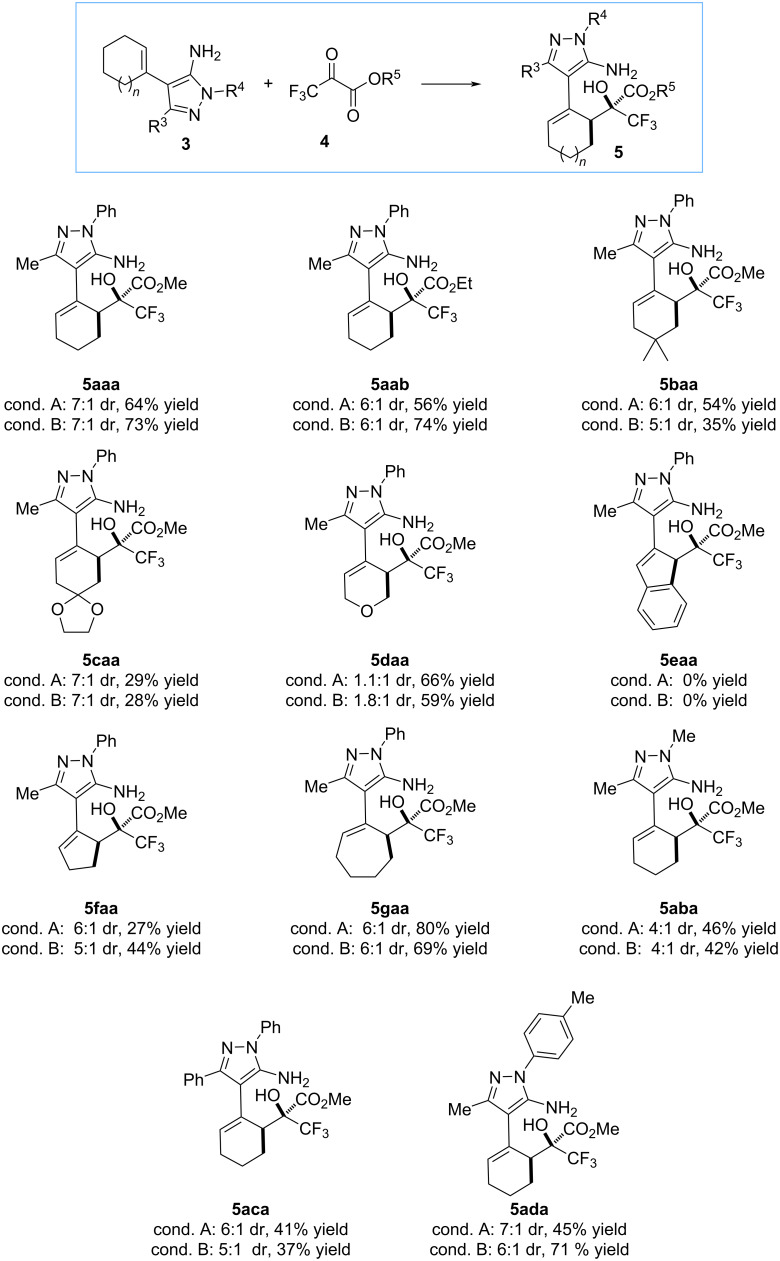
Scope of the reaction. Reaction conditions A: **3** (0.2 mmol) and **4** (0.6 mmol) in 2 mL of toluene at 70 °C. Reaction conditions B: **3** (0.2 mmol), **4** (0.6 mmol), and SQ-1 (10 mol %) in 2 mL of toluene at 50 °C. Yields refer to isolated yields after column chromatography. Diastereoisomeric ratio (dr) determined by ^1^H NMR of the crude reaction mixture.

With the optimized reaction conditions in hand, the scope of the vinylogous addition reaction of 4-alkenyl-5-aminopyrazoles **3** to alkyl trifluoropyruvates **4** was studied (Scheme 2). First, we evaluated the influence of the alkyl group in carbonyl compound **4**, where we observed similar results in terms of yield and diastereoselectivity, when methyl or ethyl trifluoropyruvate were used as reactants. Next, we tested the influence of different substituents on the cyclohexenyl ring of the aminopyrazole **3**, observing lower yields for homoallylic alcohols **5baa** and **5caa** in both conditions used. Next, we evaluated the 5-aminopyrazole **3da** prepared from tetrahydro-4*H*-pyran-4-one, and interestingly the corresponding trifluoromethyl carbinol **5daa** was afforded**,** under both reaction conditions, in good yields (66% and 59% yield, respectively) but with a very low diastereoisomeric ratio (near to 1:1). Later, we evaluated the effect of the carbocyclic ring size, observing a high yield (up to 80%) when the 5-aminopyrazole **3ga** prepared from cycloheptanone was used, while 5-aminopyrazole **3fa** bearing a cyclopentenyl ring afforded alcohol **5faa** with lower yield (27–44% yield). Unfortunately, in the case of the starting material **3ea**, prepared from 2-indanone, the corresponding product was not observed, probably due to an increase in steric hindrance. Finally, 4-cyclohexenyl-5-aminopyrazoles bearing different substituents at the N-1 or C-3 position afforded the corresponding trifluoromethyl carbinols **5aba**–**5ada** with good diastereoselectivity (4:1 to 7:1) but moderate yields (around 40%). Comparing the results of the reactions with and without **SQ-1**, we can observe that the yields improved in some cases but decreased in others, while the diastereoselectivity remained unchanged. Therefore, it is difficult to determine the role of the squaramide.

**Scheme 3:**
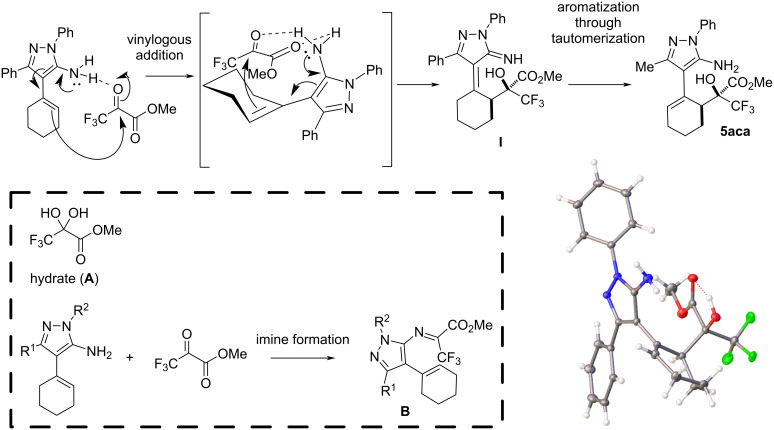
Plausible mechanism and X-ray structure of compound **5aca**.

The relative configuration of the stereogenic centres in compound **5aca** was determined by X-ray crystallographic analysis (Scheme 3) [33]; the relative configurations of the other compounds **5** were assigned on the assumption of a uniform mechanistic pathway. Considering the high diastereoselectivity observed both in the presence and absence of the squaramide catalyst, we propose a plausible mechanism (Scheme 3) that involves hydrogen bonding activation of the methyl trifluoropyruvate by the NH₂ group of the aminopyrazole. This interaction directs the attack of the double bond to the carbonyl in a relative *re*-*si* approach, generating intermediate **I**, which undergoes a tautomerization to recover the aromatic pyrazole ring. The increased yield observed in some cases with **SQ-1** may be attributed to the formation of additional hydrogen bonds that enhance electrophile activation. In certain reactions, we isolated compound **A**, the hydrate of methyl trifluoropyruvate. We hypothesized that preventing the formation of this byproduct could improve the reaction yield by using molecular sieves (entries 17 and 18, Table 1). However, when molecular sieves were added, the yield decreased, possibly due to the formation of imine **B**, which might be favored by the dehydration effect of the molecular sieves.

## Conclusion

In summary, a regioselective and diastereoselective vinylogous addition reaction of 4-alkenyl-5-aminopyrazoles to alkyl trifluoropyruvate has been studied. Several homoallylic trifluoromethyl carbinols functionalized with a pyrazole moiety were obtained under mild reaction conditions (27–80% yield). This methodology provides a straightforward access to an unprecedented class of trifluoromethyl carbinol derivatives offering a new synthetic approach to functionalize 5-aminopyrazoles.

## Supporting Information

File 1 Detailed experimental procedures, characterization data, and copies of ^1^H NMR and ^13^C NMR spectra.

## Data Availability

All data that supports the findings of this study is available in the published article and/or the supporting information of this article.
