# Supplementary material for: Vinylogous functionalization of 4-alkylidene-5-aminopyrazoles with methyl trifluoropyruvates
Source: Beilstein J Org Chem. 2025 Mar 10;21:533–40. doi: 10.3762/bjoc.21.41 (PMC11897652; doi:10.3762/bjoc.21.41)
Supplement: File 1 — Detailed experimental procedures, characterization data, and copies of 1H NMR and 13C NMR spectra. [file Beilstein_J_Org_Chem-21-533-s001.pdf]

BEILSTEIN JOURNAL OF ORGANIC CHEMISTRY

Supporting Information

for

Vinylogous functionalization of 4-alkylidene-5-aminopyrazoles with methyl trifluoropyruvates

Judit Hostalet-Romero, Laura Carceller-Ferrer, Gonzalo Blay, Amparo Sanz-Marco, José R. Pedro and Carlos Vila

Beilstein J. Org. Chem. **2025,** *21,* 533–540. doi:10.3762/bjoc.21.41

Detailed experimental procedures, characterization data, and copies of ^{1}H NMR and ^{13}C NMR spectra

License and Terms: This is a supporting information file under the terms of the Creative Commons Attribution License (https://creativecommons.org/licenses/by/4.0). Please note that the reuse, redistribution and reproduction in particular requires that the author(s) and source are credited and that individual graphics may be subject to special legal provisions.
The license is subject to the *Beilstein Journal of Organic Chemistry* terms and conditions: (https://www.beilstein-journals.org/bjoc/terms)

Table of contents

1. General experimental methods S1

2. Experimental procedures S2

i) Synthesis of 4-alkenyl-5-aminopyrazoles **3**
ii) Procedure A for the vinylogous addition of 4-alkenyl-5-aminopyrazoles **3** to alkyl trifluoropyruvates **4**.
iii) Procedure B for the vinylogous addition of 4-alkenyl-5-aminopyrazoles **3** to alkyl trifluoropyruvates **4**.
iv) Reaction of acetone with 5-aminopyrazole **2a**

3. Characterization of the 4-alkenyl-5-ainopyrazoles **3** S4

4. Characterization of products **5** S8

5. Characterization of products **7** S13

6. Asymmetric reactions...S13

7. NMR data (^{1}H NMR and ^{13}C NMR experiments) S14

8. HPLC data S36

9. References...S37

1. General experimental methods

Unless otherwise noticed, commercial reagents were used without any extra purification. Reactions were followed by thin layer chromatography (TLC) using Merck Silica Gel 60 F-254 thin layer plates. For product purification, flash column chromatography using Merck silica gel 60, 0.040–0.063 mm was used. The diastereomeric ratio (dr) was determined via ^{1}H-NMR analysis. NMR spectra were carried out in a Bruker Avance III HD spectrometer at 300 MHz for ^{1}H and at 75 MHz for ^{13}C, in a Bruker AV400 spectrometer at 400 MHz for ^{1}H and at 100 MHz for ^{13}C or in a Bruker Neo500 spectrometer at 500 MHz for ^{1}H and at 126 MHz for ^{13}C using residual non-deuterated solvent as internal standard ($CHCl_3$: δ 7.26 for ^{1}H and 77.0 ppm for ^{13}C). Chemical shifts are given in ppm. The carbon type was determined by Distortionless Enhancement by Polarization Transfer (DEPT) experiments. High resolution mass spectra (ESI) were recorded on a TRIPLETOFT5600 spectrometer equipped with an electrospray source with a capillary voltage of 4.5 kV. Cyclic ketones **1**, 5-aminopyrazoles **2** and alkyl trifluoropyruvates **4** are commercial reactants and were used without subsequent purification. These reactions have been acquired from the following commercial cases: Aldrich, TCI, BLD and Fluorochem.

2. Experimental procedures

i. Synthesis of 4-alkenyl-5-aminopyrazoles 3

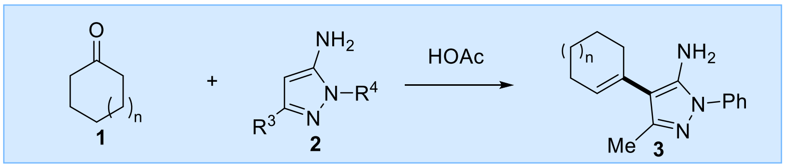

In a 25 mL round-bottomed flask, the 5-aminopyrazole (1 equiv) was dissolved in the cyclic ketone (1.5 equiv) and 0.5 mL glacial acetic acid (if the cyclic ketone was solid 2 mL of CH_2Cl_2 were used as solvent). Next, the reaction mixture was left, with stirring, at room temperature for a period of 3–5 days following the reaction using thin layer chromatography (TLC). The reaction mixture was evaporated under reduced pressure and the crude was purified via column chromatography with hexane:EtOAc mixtures as eluent to afford the corresponding products **3**.

ii. Procedure A for the vinylogous addition of 4-alkenyl-5-aminopyrazoles 3 to alkyl trifluoropyruvates 4.

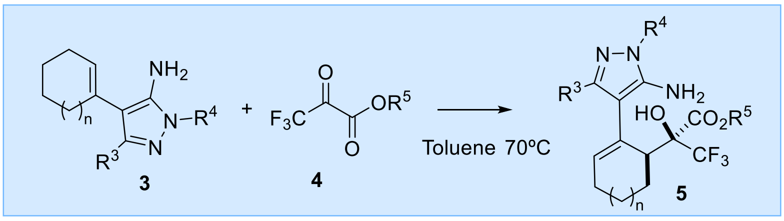

In a 10 mL round-bottomed flask, the corresponding 4-alkenyl-5-aminopyrazole (**3**, 0.2 mmol, 1 equiv) and the alkyl trifluoropyruvate (**4**, 0.6 mmol, 3 equiv) were dissolved in 2 mL toluene. The reaction mixture was heated at 70 °C with a condenser for 1 day. After this time, the solvent was evaporated under reduced pressure and the diastereoisomeric ratio was evaluated using ^{1}H NMR of the crude reaction mixture. Subsequently, the crude was purified via column chromatography with hexane:EtOAc mixtures or hexane:DCM mixtures as eluent to afford the corresponding alcohols **5**.

iii. Procedure B for the vinylogous addition of 4-alkenyl-5-aminopyrazoles 3 to alkyl trifluoropyruvates 4.

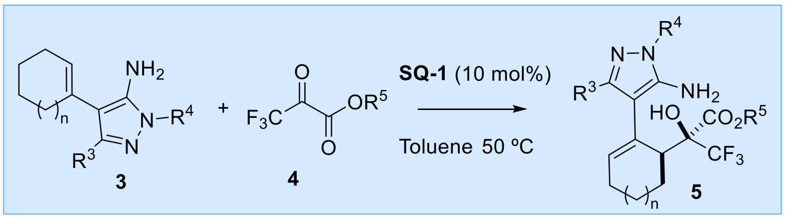

In a 10 mL round-bottomed flask, the corresponding 4-alkenyl-5-aminopyrazole (**3**, 0.2 mmol, 1 equiv), **SQ-1** (0.02 mmol, 10 mol %) and the alkyl trifluoropyruvate (**4**,

0.6 mmol, 3 equiv) were dissolved in 2 mL toluene. The reaction mixture was heated at 50 °C with a condenser for 1 day. After this time, the solvent was evaporated under reduced pressure and the diastereoisomeric ratio was evaluated using ^{1}H NMR of the crude reaction mixture. Subsequently, the crude was purified via column chromatography with hexane:EtOAc mixtures or hexane:DCM mixtures as eluent to afford the corresponding alcohols **5**.

iv. Reaction of acetone with 5-aminopyrazole 2a.

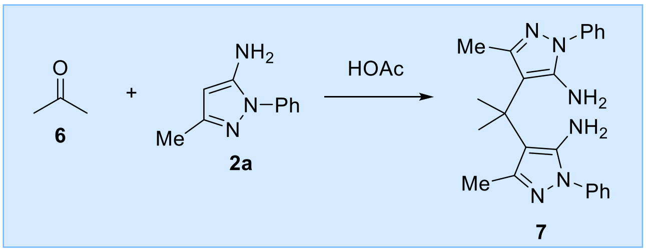

In a 25 mL round-bottomed flask, the 5-aminopyrazole **2a** (0.87 g, 5 mmol) was dissolved in acetone (0.73 mL, 10 mmol) and 0.5 mL glacial acetic acid. Next, the reaction mixture is left, with stirring, at room temperature for a period of 3–5 days following the reaction using thin layer chromatography (TLC). The reaction mixture was evaporated under reduced pressure and the crude was purified via column chromatography with hexane:EtOAc mixtures as eluent to afford the corresponding products **7**.

3. Chacterization of 4-alkenyl-5-aminopyrazoles 3

Compound **3aa**[1]:

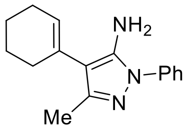

Following general procedure i), product **3aa** was synthetized using 1.2 mL (12 mmol) of cyclohexanone (**1a**) with 1.39 g (8 mmol) 5-aminopyrazole (**2a**) obtaining 0.98 g (63% yield) of the product **3aa** as a yellow solid (m.p. = 86-90 °C).

^{1}H RMN (300 MHz, CDCl$_3$) δ 7.57 (dd, *J* = 8.6, 1.2 Hz, 2H), 7.49-7.41 (m, 2H), 7.34-7.27 (m, 1H), 5.67 (tt, *J* = 3.6, 1.7 Hz, 1H), 3.77 (s, 2H), 2.30-2.13 (m, 7H), 1.82-1.60 (m, 4H). **^{13}C RMN (75 MHz, CDCl$_3$)** δ 147.2 (C), 141.6 (C), 138.9 (C), 130.5 (C), 129.4 (CH), 126.8 (CH), 126.2 (CH), 123.5 (CH), 107.1 (C), 29.2 (CH$_2$), 25.6 (CH$_2$), 23.1 (CH$_2$), 22.2 (CH$_2$), 13.6 (CH$_3$).

· Compound **3ba**:

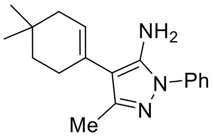

Following general procedure i), product **3ba** was synthetized using 1.51 g (12 mmol) of 4,4-dimethylcyclohexan-1-one (**1b**) with 1.39 g (8 mmol) 5-aminopyrazole (**2a**) obtaining 1.06 g (47% yield) of the product **3ba** as a yellow solid (m.p. = 123-126 °C).

^{1}H NMR (300 MHz, CDCl$_3$) δ 7.57 (dd, *J* = 8.6, 1.2 Hz, 2H), 7.53-7.40 (m, 2H), 7.35-7.27 (m, 1H), 5.59 (tt, *J* = 3.8, 1.8 Hz, 1H), 3.75 (s, 2H), 2.27 (ddt, *J* = 6.5, 4.3, 2.1 Hz, 2H), 2.23 (s, 3H), 1.98 (dd, *J* = 4.1, 2.3 Hz, 2H), 1.50 (t, *J* = 6.4 Hz, 2H), 0.99 (s, 6H). **^{13}C NMR (75 MHz, CDCl$_3$)** δ 147.2 (C), 141.6 (C), 138.9 (C), 129.4 (CH), 129.2 (C), 126.8 (CH), 125.2 (CH), 123.5 (CH), 106.8 (C), 39.6 (CH$_2$), 35.8 (CH$_2$), 28.3 (CH$_3$), 28.2 (C), 27.0 (CH$_2$), 13.6 (CH$_3$)

· Compound **3ca**:

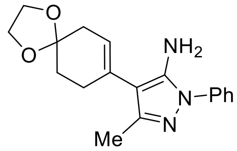

Following general procedure i), product **3ca** was synthetized using 0.94 g (6 mmol) of 1,4-dioxaspiro[4.5]decan-8-one (**1c**) with 0.69 g (4 mmol) 5-aminopyrazole (**2a**) obtaining 0.86 g (69% yield) of the product **3ca** as a yellow solid (m.p. = 102-105 °C).

^{1}H NMR (300 MHz, CDCl$_3$) δ 7.56 (dd, *J* = 8.6, 1.2 Hz, 1H), 7.51-7.39 (m, 1H), 7.34-7.27 (m, 1H), 5.56 (tt, *J* = 3.6, 1.5 Hz, 1H), 4.02 (s, 3H), 3.80 (s, 1H), 2.50 (tt, *J* = 6.0, 1.7 Hz, 1H), 2.47-2.43 (m, 1H), 2.24 (s, 2H), 1.89 (t, *J* = 6.4 Hz, 1H). **^{13}C NMR (100 MHz, CDCl$_3$)** δ 147.1 (C), 141.7 (C), 138.8 (C), 130.2 (C), 129.4 (CH), 126.9 (CH), 123.6 (CH), 122.8 (CH), 107.6 (C), 105.9 (C), 64.4 (CH$_2$), 36.1 (CH$_2$), 31.4 (CH$_2$), 28.4 (CH$_2$), 13.7 (CH$_3$).

·Compound **3da**:

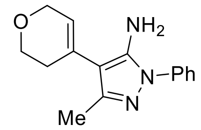

Following general procedure i), product **3da** was synthetized using 0.56 mL (6 mmol) of tetrahydro-4*H*-pyran-4-one (**1d**) with 0.69 g (4 mmol) 5-aminopyrazole (**2a**) obtaining 0.12 g (11% yield) of the product **3da** as a yellow solid (m.p. = 98-102 °C).

^{1}H NMR (300 MHz, $CDCl_3$) δ 7.57-7.48 (m, 2H), 7.42 (t, *J* = 7.8 Hz, 2H), 7.33-7.25 (m, 1H), 5.65 (tt, *J* = 2.9, 1.6 Hz, 1H), 4.27 (q, *J* = 2.7 Hz, 2H), 3.88 (t, *J* = 5.4 Hz, 2H), 3.84 (s, 2H), 2.38 (ttd, *J* = 5.4, 2.6, 1.7 Hz, 2H), 2.22 (s, 3H). **^{13}C NMR (126 MHz, $CDCl_3$)** δ 146.8 (C),141.8(C), 138.5 (C), 129.3 (CH), 128.4 (C), 126.9 (CH), 123.5 (CH), 123.5 (CH), 104.8(C), 65.5 (CH_2), 64.3 (CH_2), 29.0 (CH_2), 13.6 (CH_3).

·Compound **3ec**:

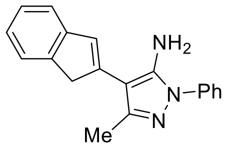

Following general procedure i), product **3da** was synthetized using 0.79 g (6 mmol) of 1,3-dihydro-2*H*-inden-2-one (**1e**) with 0.69 g (4 mmol) 5-aminopyrazole (**2a**) obtaining 0.54 g (47% yield) of the product **3da** as a brown oil.

^{1}H NMR (300 MHz, $CDCl_3$) δ 7.58 (dd, *J* = 8.7, 1.2 Hz, 2H), 7.54-7.46 (m, 2H), 7.47 (dd, *J* = 7.3, 0.9 Hz, 1H), 7.41-7.34 (m, 2H), 7.28 (dd, *J* = 7.5, 1.2 Hz, 1H), 7.15 (td, *J* = 7.3, 1.1 Hz, 1H), 6.79-6.77 (m, 1H), 4.12 (s, 2H), 3.79 (s, 2H), 2.45 (s, 3H). **^{13}C NMR (126 MHz, $CDCl_3$)** δ 147.5 (C), 145.5 (C), 142.8 (C), 141.9 (C), 139.9 (C), 138.2 (C), 129.6 (CH), 127.5 (CH), 126.6 (CH), 124.0 (CH), 123.8 (CH), 123.7 (CH), 123.3 (CH), 120.0 (CH), 101.2 (C), 41.1 (CH_2), 14.9 (CH_3)

·Compound **3fa**:

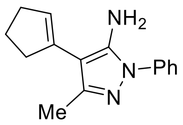

Following general procedure i), product **3da** was synthetized using 1.1 mL (12 mmol) of cyclopentanone (**1f**) with 1.39 g (8 mmol) 5-aminopyrazole (**2a**) obtaining 0.10 g (5% yield) of the product **3fa** as a yellow oil.

^{1}H NMR (300 MHz, $CDCl_3$) δ 7.35 (dd, *J* = 7.4, 1.2 Hz, 2H), 7.32 – 7.20 (m, 2H), 7.18 – 7.06 (m, 1H), 5.45 (p, *J* = 2.3 Hz, 1H), 3.75 (s, 2H), 2.58 – 2.45 (m, 2H), 2.38 – 2.24 (m, 2H), 2.11 (s, 3H), 1.86 – 1.70 (m, 2H). **^{13}C NMR (101 MHz, $CDCl_3$)** δ 147.4 (C), 142.3(C), 138.5 (C), 135.6 (C), 129.4 (CH), 127.0 (CH), 123.7(CH), 123.7(CH), 101.4(C), 35.3(CH_2), 32.6(CH_2), 23.4(CH_2), 14.4 (CH_3).

· Compound **3ga**:

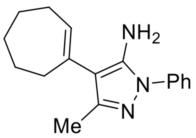

Following general procedure i), product **3ga** was synthetized using 1.4 mL (12 mmol) of cycloheptanone (**1g**) with 1.39 g (8 mmol) 5-aminopyrazole (**2a**) obtaining 0.12 g (6% yield) of the product **3ga** as a yellow oil.

^{1}H NMR (400 MHz, $CDCl_3$) δ 7.56 (d, *J* = 7.5 Hz, 2H), 7.44 (t, *J* = 7.8 Hz, 2H), 7.31 (dd, *J* = 7.4, 1.1 Hz, 1H), 5.84 (t, *J* = 6.6 Hz, 1H), 3.79 (s, 2H), 2.61 – 2.37 (m, 2H), 2.32 – 2.25 (m, 2H), 2.23 (d, *J* = 1.0 Hz, 3H), 1.84 (dtd, *J* = 11.8, 5.6, 3.2 Hz, 2H), 1.73 – 1.50 (m, 4H). **^{13}C NMR (101 MHz, $CDCl_3$)** δ 146.9 (C), 141.3 (C), 138.7 (C), 137.2 (C), 131.5 (CH), 129.4 (CH), 126.9 (CH), 123.5 (CH), 108.9 (C), 33.9 (CH_2), 32.8 (CH_2), 28.8 (CH_2), 27.3 (CH_2), 27.2 (CH_2), 13.3 (CH_3).

· Compound **3ab[2]**:

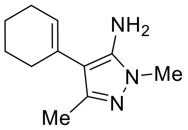

Following general procedure i), product **3aa** was synthetized using 0.6 mL (6 mmol) of cyclohexanone (**1a**) with 0.44 g (8 mmol) 5-aminopyrazole (**2b**) obtaining 0.41 g (54% yield) of the product **3ab** as a white solid (m.p. = 178-180 °C).

^{1}H NMR (400 MHz, $CDCl_3$) 5.54 (tt, *J* = 3.5, 1.6 Hz, 1H), 3.60 (s, 3H), 3.45 (s, 1H), 2.22 – 2.15 (m, 4H), 2.14 (s, 3H), 1.75-1.61 (m, 4H). **^{13}C NMR (75 MHz, $CDCl_3$)** δ 144.7 (C), 141.5 (C), 130.9 (C), 125.5 (CH), 107.4 (C), 34.1 (CH_3), 29.3 (CH_2), 25.5 (CH_2), 23.1 (CH_2), 22.2 (CH_2), 13.5 (CH_3).

· Compound **3ac[2]**:

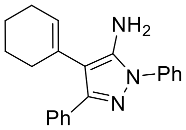

Following general procedure i), product **3ac** was synthetized using 0.3 mL (3 mmol) of cyclohexanone (**1a**) with 0.47 g (2 mmol) 5-aminopyrazole (**2c**) obtaining 0.10 g (16% yield) of the product **3ac** as an orange solid (m.p. = 96-106 °C).

^{1}H NMR (400 MHz, $CDCl_3$) δ 7.81 – 7.74 (m, 2H), 7.69 (dd, *J* = 8.6, 1.2 Hz, 2H), 7.53 – 7.44 (m, 2H), 7.41 – 7.34 (m, 2H), 7.34 – 7.27 (m, 2H), 5.84 (tt, *J* = 3.7, 1.7 Hz, 1H), 3.84 (s, 2H), 2.25 (d, *J* = 2.3 Hz, 2H), 2.03 (d, *J* = 1.9 Hz, 2H), 1.68 (t, *J* = 3.2 Hz, 4H). **^{13}C NMR (75 MHz, $CDCl_3$)** δ 149.0 (C), 142.3 (C), 134.0 (C), 134.3 (C), 131.4 (C), 129.4 (CH), 128.1 (CH), 127.49 (CH), 127.46 (CH), 127.2 (CH), 127.0 (CH), 123.6 (CH), 106.4 (C), 29.4 (CH_2), 25.8 (CH_2), 23.1 (CH_2), 22.2 (CH_2).

· Compound **3ad**:

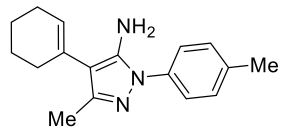

Following general procedure i), product **3ac** was synthetized using 0.3 mL (3 mmol) of cyclohexanone (**1a**) with 0.37 g (2 mmol) 5-aminopyrazole (**2d**) obtaining 0.25 g (46% yield) of the product **3ac** as a yellow solid (m.p. = 91-92 °C).

^{1}H NMR (300 MHz, $CDCl_3$) δ 7.43 (d, *J* = 8.4 Hz, 2H), 7.31 – 7.11 (m, 2H), 5.66 (tt, *J* = 3.6, 1.7 Hz, 1H), 3.73 (s, 2H), 2.38 (s, 3H), 2.29 – 2.11 (m, 7H), 1.88 – 1.59 (m, 4H). **^{13}C NMR (126 MHz, $CDCl_3$)** δ 146.8 (C), 141.6 (C), 136.7 (C), 136.4 (C), 130.6 (C), 129.9 (CH), 126.0 (CH), 123.6 (CH), 106.9 (C), 29.3 (CH_2), 25.6 (CH_2), 23.1 (CH_2), 22.3 (CH_2), 21.1 (CH_3), 13.6 (CH_3).

4. Characterization of products 5

· Compound **5aaa**:

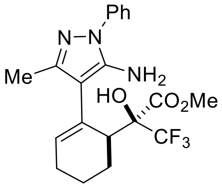

Following general procedure A, product **5aaa** was synthetized as colorless oil in 64% yield (0.128 mmol, 52.4 mg). Diastereoisomeric ratio (7:1) was determined via ^{1}H NMR analysis. The enantiomeric excess was determined by HPLC using a chiral stationary phase (Chiralpak® AYH), using hexane:iPrOH 80:20, 1.0mL/min, first enantiomer t_R = 4.77 min, second enantiomer t_R = 6.17 min.

^{1}H NMR (300 MHz, CDCl$_3$) δ 7.58-7.51 (m, 2H), 7.46 (t, *J*= 7.7 Hz, 2H), 7.37-7.29 (m, 1H), 5.89 (td, *J* = 3.9, 1.4 Hz, H), 4.06 (s, 1H), 3.74 (s, 2H), 3.54 (s, 3H), 3.38-3.30 (m, 1H), 2.19 (ddd, *J* = 7.7, 3.7, 1.9 Hz, 2H), 2.13 (s, 3H), 2.10-1.88 (m, 3H), 1.74-1.52 (m, 1H). **^{13}C NMR (75 MHz, CDCl$_3$)** δ 170.6 (C), 147.5 (C), 143.1 (C), 138.7 (C), 135.5 (CH), 129.5 (CH), 127.2 (CH), 126.4 (C), 123.8 (CH), 123.72 (q, *J* = 288.8 Hz, CF$_3$), 104.5 (C), 79.5 (q, *J* = 27.4 Hz, C), 53.8 (CH$_3$), 40.7 (CH), 25.5 (CH$_2$), 24.7 (q, *J* = 2.5 Hz, CH$_2$), 19.31 (CH$_2$), 12.64 (CH$_3$). **^{19}F NMR (282 MHz, CDCl$_3$)** δ -73.51. **HRMS (ESI)** m/z 410.1689 [M+H]$^+$ $C_{20}H_{23}F_3N_3O_3^+$ requires 410.1686.

· Compound **5aab**:

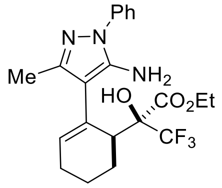

Following general procedure A, product **5aab** was synthetized as colorless oil in 56% yield (0.112 mmol, 50.9 mg). Diastereoisomeric ratio (6:1) was determined via ^{1}H NMR analysis.

^{1}H NMR (500 MHz, CDCl$_3$) δ 7.52 (dd, *J* = 8.6, 1.2 Hz, 2H), 7.49-7.41 (m, 2H), 7.32 (tt, *J* = 7.0, 1.3 Hz, 1H), 5.88 (td, *J* = 3.9, 1.3 Hz, 1H), 4.10-4.01 (m, 2H), 3.76-3.63 (m, 3H), 3.33 (s, 1H), 2.27-2.16 (m, 2H), 2.13 (s, 3H), 2.12-2.06 (m, 1H), 2.03-1.96 (m, 1H), 1.95-1.82 (m, 1H), 1.63 (dtd, *J* = 13.0, 6.8, 2.8 Hz, 1H), 1.21 (t, *J* = 7.2 Hz, 3H). **^{13}C NMR (75 MHz, CDCl$_3$)** δ 170.1 (C), 147.5 (C), 143.2 (C), 138.6 (C), 135.4 (CH), 129.5 (CH), 127.2 (CH), 126.4 (C), 123.77 (CH), 123.77 (q, *J* = 288.9 Hz, CF$_3$), 104.6 (C), 79.6 (q, *J* = 27.4 Hz), 63.6 (CH2), 40.5 (CH), 25.5 (CH$_2$), 24.7 (q, *J* = 2.4 Hz, CH$_2$), 19.1 (CH$_2$), 13.5 (CH$_3$), 12.7 (CH$_3$). **^{19}F NMR (282 MHz, CDCl$_3$)** δ -73.04. **HRMS (ESI)** m/z 424.1847 [M+H]$^+$ $C_{21}H_{25}F_3N_3O_3^+$ requires 424,1843.

·

Compound **5baa**:

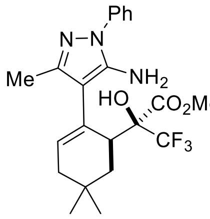

Following general procedure A, product **5baa** was synthetized as colorless oil in 54% yield (0.108 mmol, 47.5 mg). Diastereoisomeric ratio (6:1) was determined via ^{1}H NMR analysis.

^{1}H NMR (300 MHz, $CDCl_3$) δ 7.54 (dd, *J* = 8.4, 1.3 Hz, 2H), 7.49-7.42 (m, 2H), 7.38-7.29 (m, 1H), 5.78 (dt, *J* = 6.1, 2.1 Hz, 1H), 4.31 (s, 1H), 3.75 (s, 2H), 3.60 (s, 3H), 3.34 (dtt, *J* = 9.7, 3.8, 2.0 Hz, 1H), 2.08 (s, 3H), 2.03 (dd, *J* = 4.1, 2.1 Hz, 1H), 1.91-1.58 (m, 3H), 1.06 (s, 3H), 1.05 (s, 3H). **^{13}C NMR (75 MHz, $CDCl_3$)** δ 170.1 (C), 147.9 (C), 143.1 (C), 138.5 (C), 135.2 (CH), 129.5 (CH), 127.6 (q, *J* = 273.5 Hz, CF_3), 127.4 (CH), 124.1 (CH), 104.0 (C), 79.8 (q, *J* = 26.9 Hz, C), 53.8 (CH_3), 40.8 (CH), 39.0 (CH_2), 36.7 (CH_2), 31.8 (CH_3), 29.2 (C), 24.3 (CH_3), 12.3 (CH_3). **^{19}F NMR (282 MHz, $CDCl_3$)** δ -72.76. **HRMS (ESI)** m/z 438.2005 $[M+H]^+$ $C_{22}H_{27}F_3N_3O_3^+$ requires 438.1999.

· Compound **5caa**:

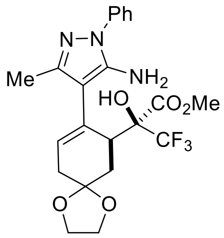

Following general procedure A, product **5caa** was synthetized as colorless oil in 29% yield (0.058 mmol, 27.2 mg). Diastereoisomeric ratio (7:1) was determined via ^{1}H NMR analysis.

^{1}H NMR (500 MHz, $CDCl_3$) δ 7.54 (dd, *J* = 8.6, 1.2 Hz, 1H), 7.48-7.42 (m, 1H), 7.35-7.29 (m, 1H), 5.81 (td, *J* = 3.9, 1.6 Hz, 1H), 5.77 (s, 1H), 4.1-3.96 (m, 4H), 3.83 (s, 2H), 3.77-3.70 (m, 1H), 3.48 (s, 3H), 2.52-2.48 (m, 1H), 2.31-2.25 (m, 1H), 2.19-2.14 (m, 1H), 2.13 (s, 2H) . **^{13}C NMR (126 MHz, $CDCl_3$)** δ 168.1 (C), 147.8 (C), 144.0 (C), 138.6 (C), 135.8 (C), 131.8 (CH), 129.5 (CH), 127.2 (CH), 126.6 (C), 123.8 (CH), 106.7 (C), 79.8 (q, *J* = 27.0 Hz, C), 64.9 (CH_2), 64.7 (CH_2), 53.1 (CH_3), 36.5 (CH_2), 32.7 (CH_2), 30.3 (CH), 12.5 (CH_3). **^{19}F NMR (282 MHz, $CDCl_3$)** δ -72.77. **HRMS (ESI)** m/z 468.1738 $[M+H]^+$ $C_{22}H_{25}F_3N_3O_5^+$ requires 468.1741.

•

Compound **5daa**:

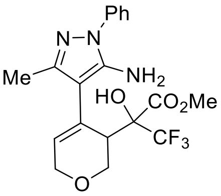

Following general procedure A, product **5daa** was synthetized as yellow oil in 66% yield (0.132 mmol, 54.2 mg). Diastereoisomeric ratio (1.13:1) was determined via ^{1}H NMR analysis.

Diastereoisomer 1: **^{1}H NMR (400 MHz, $CDCl_3$)** δ 7.52 (dd, *J* = 8.6, 1.3 Hz, 2H), 7.50-7.43 (m, 2H), 7.37-7.30 (m, 1H), 5.96 (dt, *J* = 2.9, 1.5 Hz, 1H), 4.70 (s, 1H), 4.45 (d, *J* = 12.5 Hz, 1H), 4.42-4.30 (m, 2H), 3.94-3.88 (m, 1H), 3.83 (d, *J* = 2.5 Hz, 2H), 3.43 (s, 3H), 3.28 (s, 1H), 2.18 (s, 3H). **^{13}C NMR (75 MHz, $CDCl_3$)** δ 167.8 (C), 147.2 (C), 143.7 (C), 138.4 (C), 130.9 (CH), 129.60 (CH), 127.3 (CH), 125.7 (C), 123.8 (q, *J* = 284.8 Hz, CF_3), 123.6 (CH), 102.4 (C), 79.80 (q, *J* = 28.0 Hz, C), 66.6 (CH_2), 66.0 (CH_2), 53.0 (CH_3), 39.5 (CH), 13.0 (CH_3). **^{19}F NMR (282 MHz, $CDCl_3$)** δ -73.62. **HRMS (ESI)** m/z 412.1482 $[M+H]^+$ $C_{19}H_{21}F_3N_3O_4^+$ requires 412.1479.

Diastereoisomer 2: **^{1}H NMR (400 MHz, $CDCl_3$)** δ 7.54 (d, *J* = 7.5 Hz, 2H), 7.46 (t, *J* = 7.6 Hz, 2H), 7.32 (t, *J* = 7.4 Hz, 1H), 5.92 (t, *J* = 2.3 Hz, 1H), 4.34 (d, *J* = 2.9 Hz, 1H), 4.32 (t, *J* = 2.3 Hz, 1H), 4.29-4.24 (m, 1H), 4.17 (d, *J* = 12.2 Hz, 1H), 3.92 (s, 2H), 3.85 (d, *J* = 1.1 Hz, 3H), 3.79 (dd, *J* = 12.2, 2.7 Hz, 1H), 3.16 (s, 1H), 2.21 (s, 3H). **^{13}C NMR (75 MHz, $CDCl_3$)** δ 169.8 (C), 146.6 (C), 142.6 (C), 138.5 (C), 129.9 (CH), 129.5 (CH), 127.2 (CH), 125.8 (C), 123.7 (CH), 104.4 (C), 80.2 (q, *J* = 28.8 Hz, C), 66.3 (CH_2), 65.8 (CH_2), 53.8 (CH_3), 40.2 (CH), 13.3 (CH_3). **^{19}F NMR (282 MHz, $CDCl_3$)** δ -74.68. **HRMS (ESI)** m/z 412.1485 $[M+H]^+$ $C_{19}H_{21}F_3N_3O_4^+$ requires 412.1479.

· Compound **5faa**:

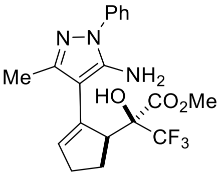

Following general procedure A, product **5faa** was synthetized as orange oil in 27% yield (0.054 mmol, 21.3 mg). Diastereoisomeric ratio (6:1) was determined via ^{1}H NMR analysis.

^{1}H NMR (300 MHz, $CDCl_3$) δ 7.53 (dd, *J* = 8.6, 1.4 Hz, 2H),7.47 (t, *J* = 7.6 Hz, 2H), 7.37-7.29 (m, 1H), 5.91 (q, *J* = 2.2 Hz, 1H), 3.93 (s, 1H), 3.78-3.69 (m, 2H), 3.50 (s, 3H), 2.57 (dddd, *J* = 17.5, 6.4, 5.1, 2.5Hz, 1H), 2.49-2.35 (m, 2H), 2.23-2.15 (m, 1H), 2.13 (s, 3H), 2.04-1.87 (m, 1H). **^{13}C NMR (75 MHz, $CDCl_3$)** δ 170.2 (C), 147.8 (C), 143.4 (C), 138.5 (C), 136.7 (CH), 131.5 (C), 129.5 (CH), 127.3 (CH), 123.8 (CH), 99.5 (C), 78.8 (q, *J* = 27.6 Hz, C), 53.7 (CH_3), 49.5 (CH), 31.9 (CH_2), 25.0 (q, *J* = 2.5 Hz, CH_2), 12.7 (CH_3). **^{19}F NMR (282 MHz, $CDCl_3$)** δ -74.65. **HRMS (ESI)** m/z 447,1526 $[M+H]^+$ $C_{19}H_{21}F_3N_3O_3^+$ requires 396.1530.

· <u>Compound **5gaa**</u>:

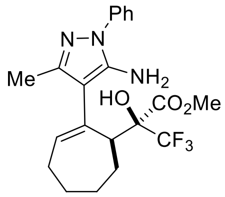

Following general procedure A, product **5gaa** was synthetized as orange oil in 80% yield (0.160 mmol, 68.0 mg). Diastereoisomeric ratio (6:1) was determined via ^{1}H NMR analysis.

^{1}H NMR (300 MHz, CDCl$_3$) δ 7.51 (dd, *J* = 8.6, 1.4 Hz, 2H), 7.48- 7.40 (m, 2H), 7.34-7.28 (m, 1H), 5.94 (dd, *J* = 8.9, 5.2 Hz, 1H), 4.02 (s, 1H), 3.71 (s, 2H), 3.46 (t, *J* = 6.3 Hz, 1H), 3.34 (s, 3H), 2.85- 2.66 (m, 1H), 2.21 (s, 3H), 2.18-1.96 (m, 3H), 1.92-1.69 (m, 3H), 1.51-1.36 (m, 1H). **^{13}C NMR (101 MHz, CDCl$_3$)** δ 171.1 (C), 147.2 (C), 142.4 (C), 138.6 (C), 136.5 (CH), 130.0 (C), 129.5 (CH), 127.0 (CH), 123.6 (q, *J* = 288.0 Hz, CF$_3$), 123.3 (CH), 107.3 (C), 80.7 (q, *J* = 28.2 Hz, C), 53.5 (CH$_3$), 47.0 (CH), 26.7 (CH$_2$), 26.4 (CH$_2$), 26.1 (CH$_2$), 24.6 (CH$_2$), 13.4 (CH$_3$). **^{19}F NMR (282 MHz, CDCl$_3$)** δ -73.52. **HRMS (ESI)** m/z 424.1849 [M+H]$^+$ $C_{21}H_{25}F_3N_3O_3^+$ requires 424.1843.

· <u>Compound **5aba**</u>:

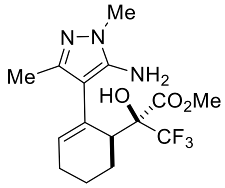

Following general procedure A, product **5aba** was synthetized as yellow solid in 46% yield (0.092 mmol, 32.2 mg) (m. p.= 99-107 °C). Diastereoisomeric ratio (4:1) was determined via ^{1}H NMR analysis.

^{1}H NMR (400 MHz, CDCl$_3$) δ 5.75 (td, *J* = 3.9, 1.3 Hz, 1H), 3.98 (s, 1H), 3.56 (s, 3H), 3.48-3.36 (m, 5H), 3.27 (tq, *J* = 5.3, 1.8 Hz, 1H), 2.21-2.12(m, 2H), 2.04 (s, 3H), 2.02-1.93 (m, 2H), 1.91-1.80 (m, 1H), 1.59 (tdq, *J* = 9.0, 5.9, 2.8 Hz, 1H). **^{13}C NMR (75 MHz, CDCl$_3$)** δ 170.7 (C), 145.3 (C), 143.1 (C), 134.9(CH),126.7 (C), 123.75 (q, *J* = 289.1 Hz, CF$_3$), 105.1 (C), 79.6 (q, *J* = 27.4 Hz, C), 53.7 (CH$_3$), 40.6 (CH), 34.1 (CH$_3$), 25.5 (CH$_2$), 24.7 (q, *J* = 2.3 Hz, CH$_2$), 19.1 (CH$_2$), 12.6 (CH$_3$). **^{19}F NMR (282 MHz, CDCl$_3$)** δ -73.55. **HRMS (ESI)** m/z 348.1531 [M+H]$^+$ $C_{15}H_{20}F_3N_3O_3^+$ requires 348.1530.

· <u>Compound **5aca**</u>:

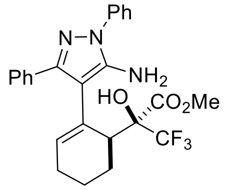

Following general procedure A, product **5aca** was synthetized as yellow solid in 41% yield (0.082 mmol, 39.3 mg) (mp 186–200 °C). Diastereoisomeric ratio (6:1) was determined via ^{1}H NMR analysis.

^{1}H NMR (300 MHz, $CDCl_3$) δ 7.84-7.77 (m, 2H), 7.64 (dd, *J* = 8.5, 1.2 Hz, 2H), 7.50 (t, *J* =7.7 Hz, 2H), 7.43-7.28 (m, 4H), 6.13 (td, *J* = 3.8, 1.1 Hz, 1H), 4.05 (s, 1H), 3.83 (s, 2H), 3.48 (s, 3H), 3.11 (s, 1H), 2.36-2.28 (m, 2H), 2.17-1.94 (m, 2H), 1.88-1.60 (m, 2H). **^{13}C NMR (126 MHz, $CDCl_3$)** δ 170.9 (C), 148.5 (C), 144.1 (C), 138.7 (C), 135.5 (CH), 133.8 (C), 129.6 (CH), 128.4 (CH), 127.8(CH), 127.6 (CH), 127.4 (C), 126.3 (CH), 124.1 (CH), 123.6 (q, *J* = 288.9 Hz, CF_3), 103.3 (C), 79.6 (q, *J* = 27.7 Hz, C), 53.9 (CH_3), 40.2 (CH), 25.8 (CH_2), 24.9 (q, *J* = 1.8 Hz, CH_2), 18.4 (CH_2). **^{19}F NMR (282 MHz, $CDCl_3$)** δ -73.71. **HRMS (ESI)** m/z 348.1531 $[M+H]^+$ $C_{15}H_{20}F_3N_3O_3^+$ requires 348.1530.

· Compound **5ada**:

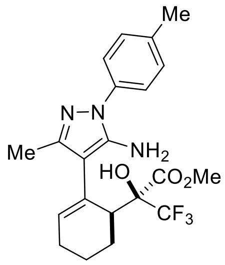

Following general procedure A, product **5ada** was synthetized as yellow solid in 45% yield (0.09 mmol, 38.2 mg) (mp 163–167 °C). Diastereoisomeric ratio (7:1) was determined via ^{1}H NMR analysis.

^{1}H NMR (300 MHz, $CDCl_3$) δ 7.39 (d, J = 8.3 Hz, 2H), 7.25 (d, J = 8.0 Hz, 2H), 5.88 (td, *J* = 3.9, 1.4 Hz, 1H), 4.07 (s, 1H), 3.70 (s, 2H), 3.53 (s, 3H), 3.37-3.30 (m, 1H), 2.39 (s, 3H), 2.19 (dd, *J* = 6.2, 3.6 Hz, 2H), 2.12 (s, 3H), 2.07-1.84 (m, 3H), 1.73-1.55 (m, 1H). **^{13}C NMR (126 MHz, $CDCl_3$)** δ 170.6 (C), 147.3 (C), 143.1 (C), 137.3 (C), 136.0 (C), 135.5 (CH), 130.1 (CH), 126.4 (C), 123.9 (CH), 123.8 (q, *J* = 288.8 Hz, CF_3), 104.3 (C), 79.7 (q, *J* = 27.3 Hz, C), 53.8 (CH_3), 40.8 (CH), 25.6 (CH_2), 24.7 (q, *J* = 2.4 Hz, CH_2), 21.1 (CH_3), 19.4 (CH_2), 12.6 (CH_3). **^{19}F NMR (282 MHz, $CDCl_3$)** δ -73.51. **HRMS (ESI)** m/z 424.1839 $[M+H]^+$ $C_{21}H_{25}F_3N_3O_3^+$ requires 424.1843.

5. Characterization of product 7

· Compound **7**:

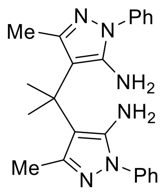

Following procedure iv, product **7** was synthetized as a colorless oil in 37% yield (0.93 mmol, 360 mg).

^{1}H NMR (300 MHz, CDCl$_3$) δ 7.50-7.38 (m, 8H), 7.35-7.25 (m, 2H), 3.61 (s, 4H), 2.34 (s, 6H), 1.72 (s, 6H). **^{13}C NMR (75 MHz, CDCl$_3$)** δ 146.4 (C), 142.4 (C), 138.4 (C), 129.4 (CH), 127.2 (CH), 124.0 (CH), 106.9 (C), 33.7 (C), 29.4 (CH$_3$), 16.1 (CH$_3$). **HRMS (ESI)** m/z 387.2300 $[M+H]^+$ $C_{23}H_{27}N_6^+$ requires 387.2292.

6. Asymmetric reactions

Scheme S.1 - Optimization of the organocatalyst.

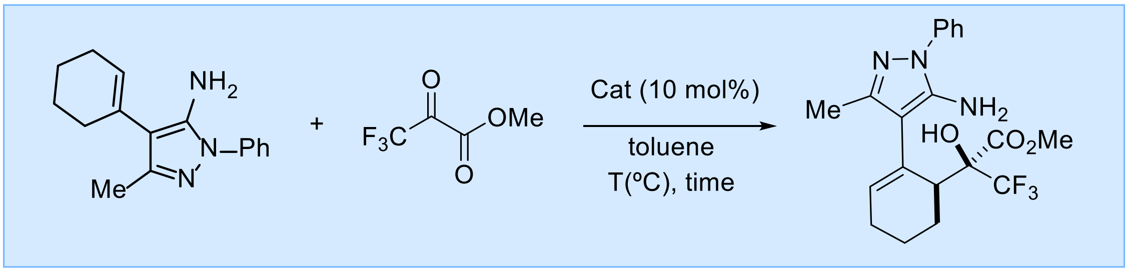

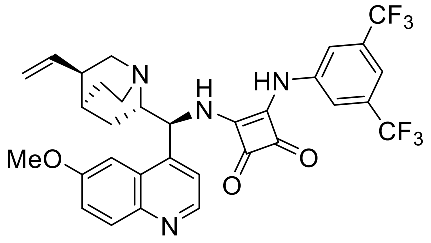

rt, 5 dies, 30% yield, 6:1 dr, 0% ee
50ºC, 1 day, 52% yield, 7:1 dr, 0% ee

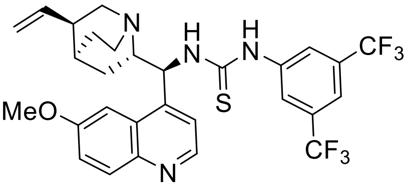

50ºC, 3 days, 58% yield, 7:1 dr, 0% ee

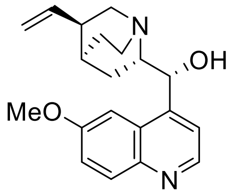

50ºC, 3 days, 51% yield,6:1 dr, 0% ee

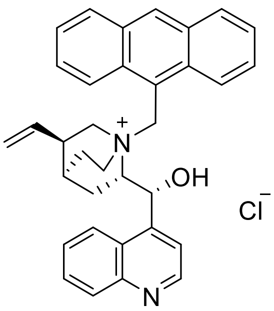

50ºC, 3 days, 55%, 7:1 dr, 0% ee

7. NMR data

· Compound **3aa**:

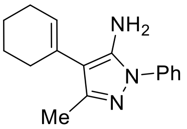

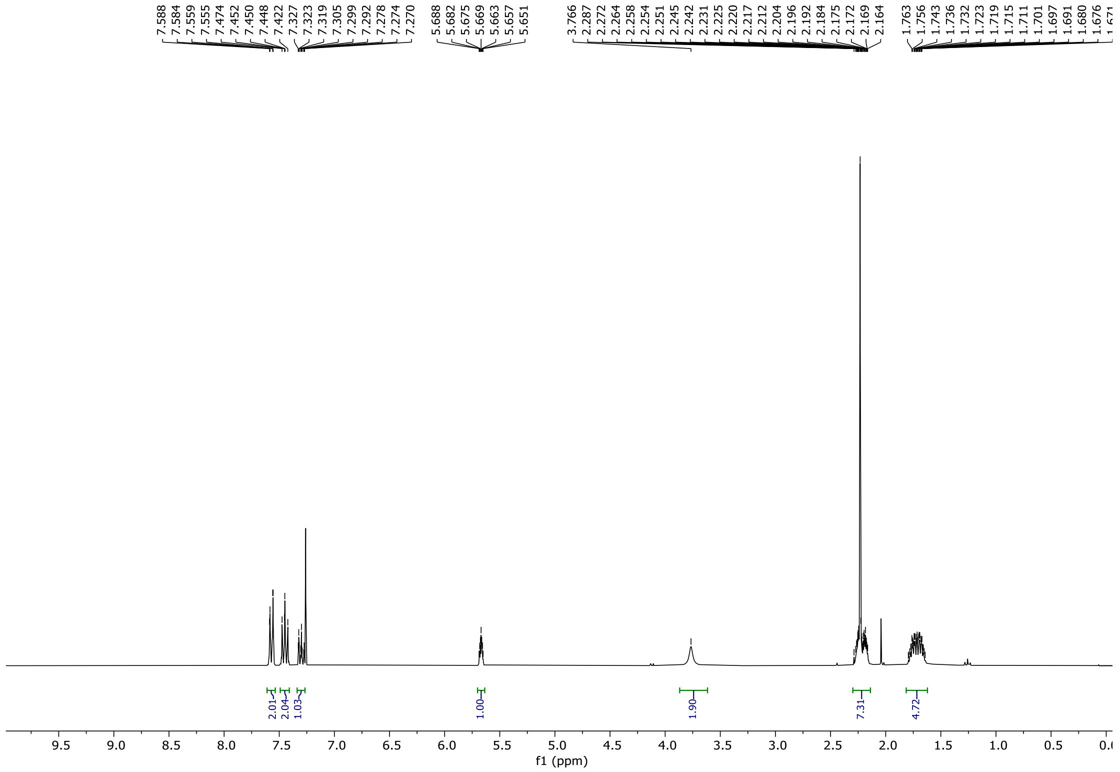

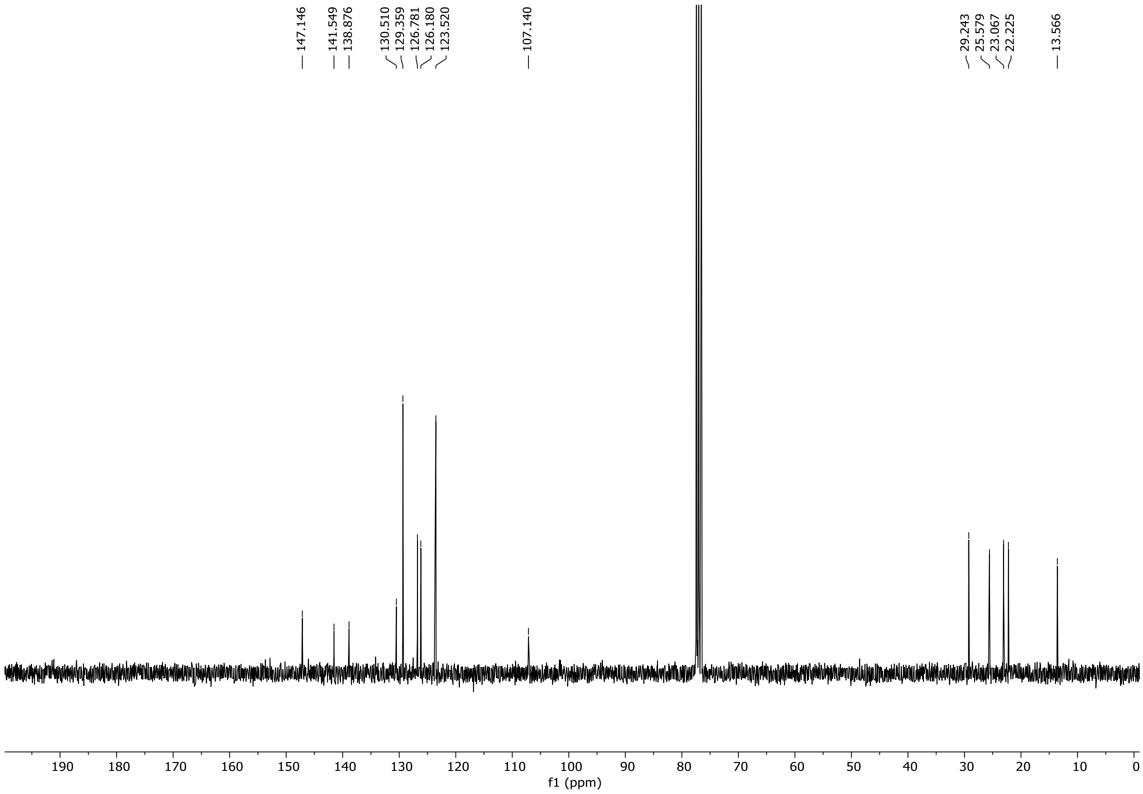

•

Compound **3ba**:

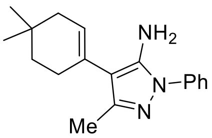

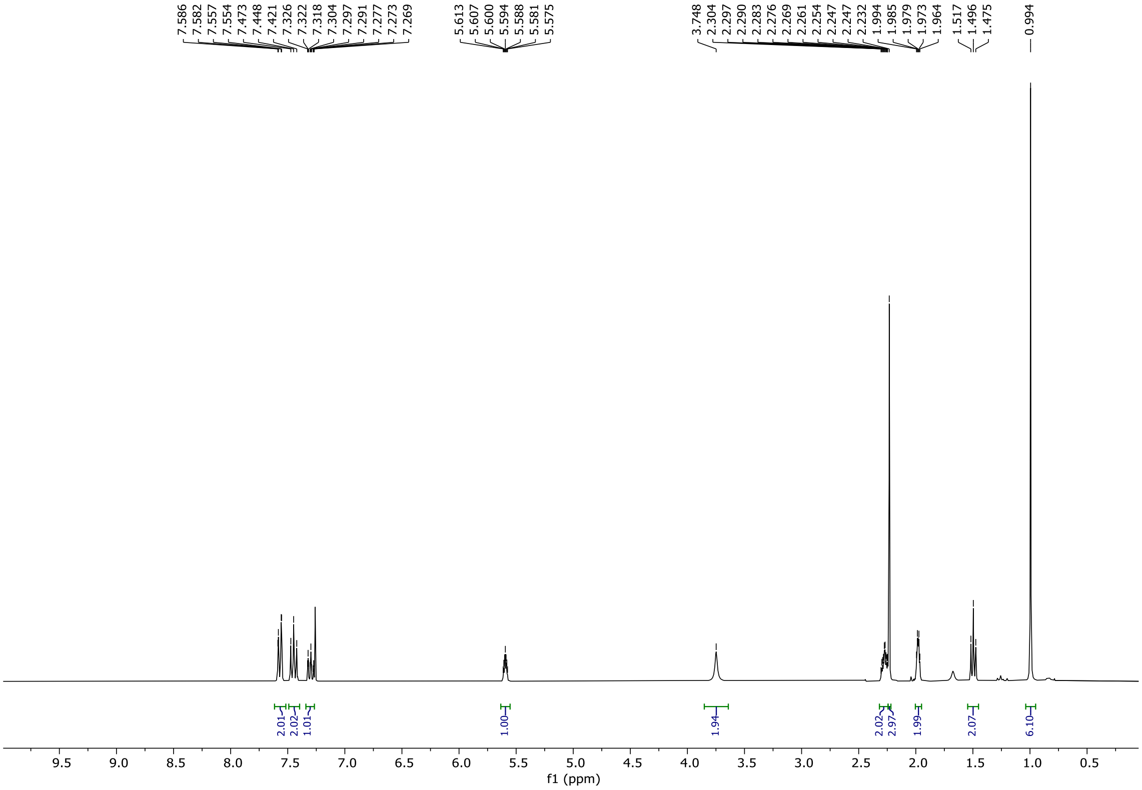

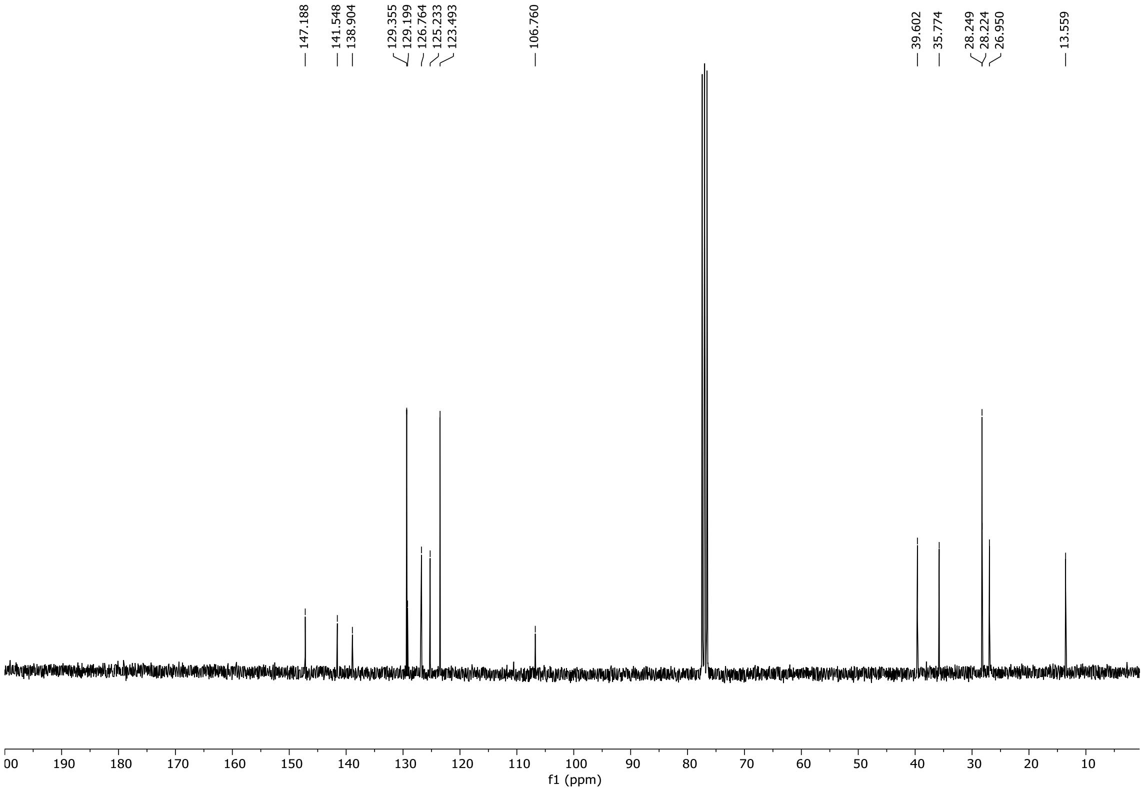

Compound **3ca**:

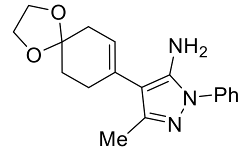

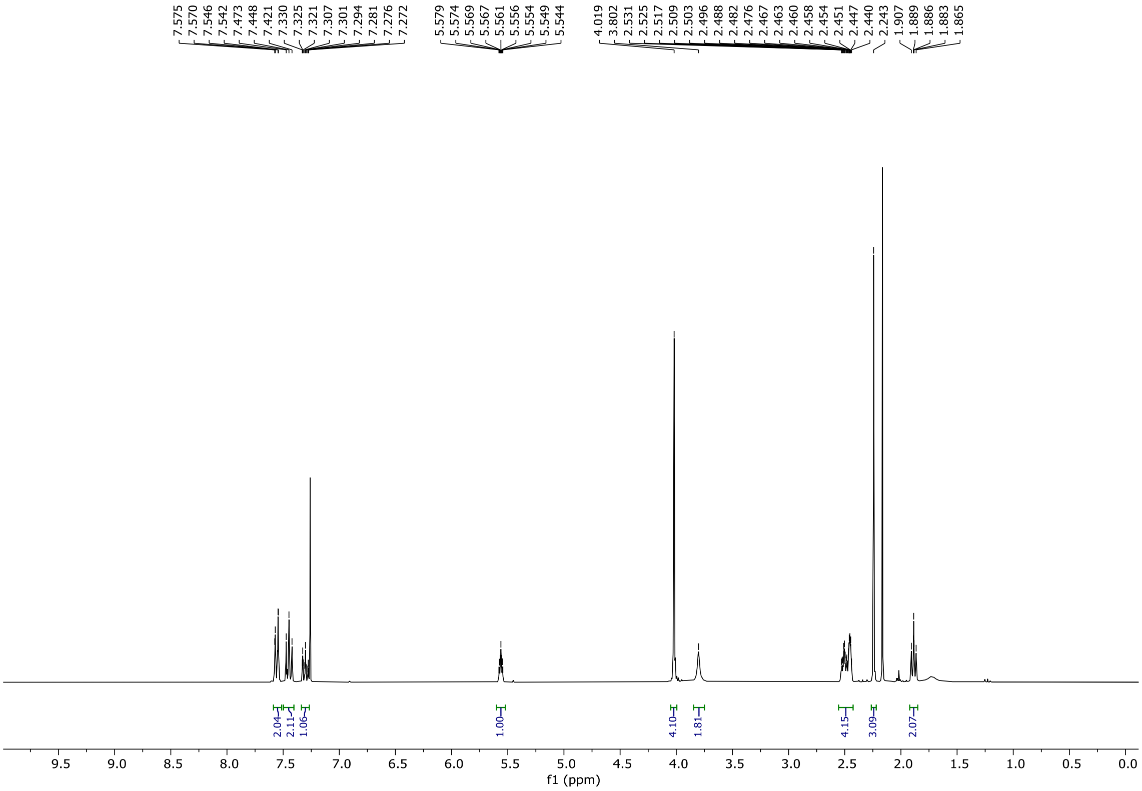

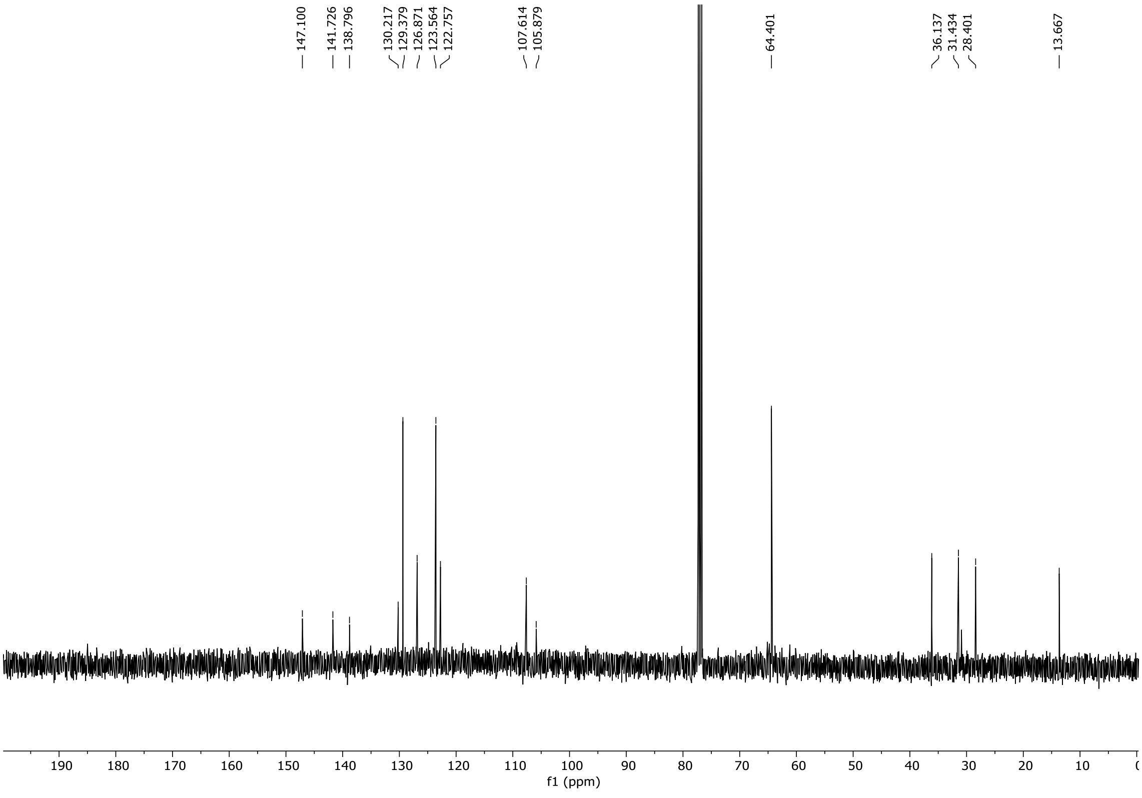

•

Compound **3da**:

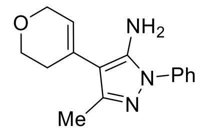

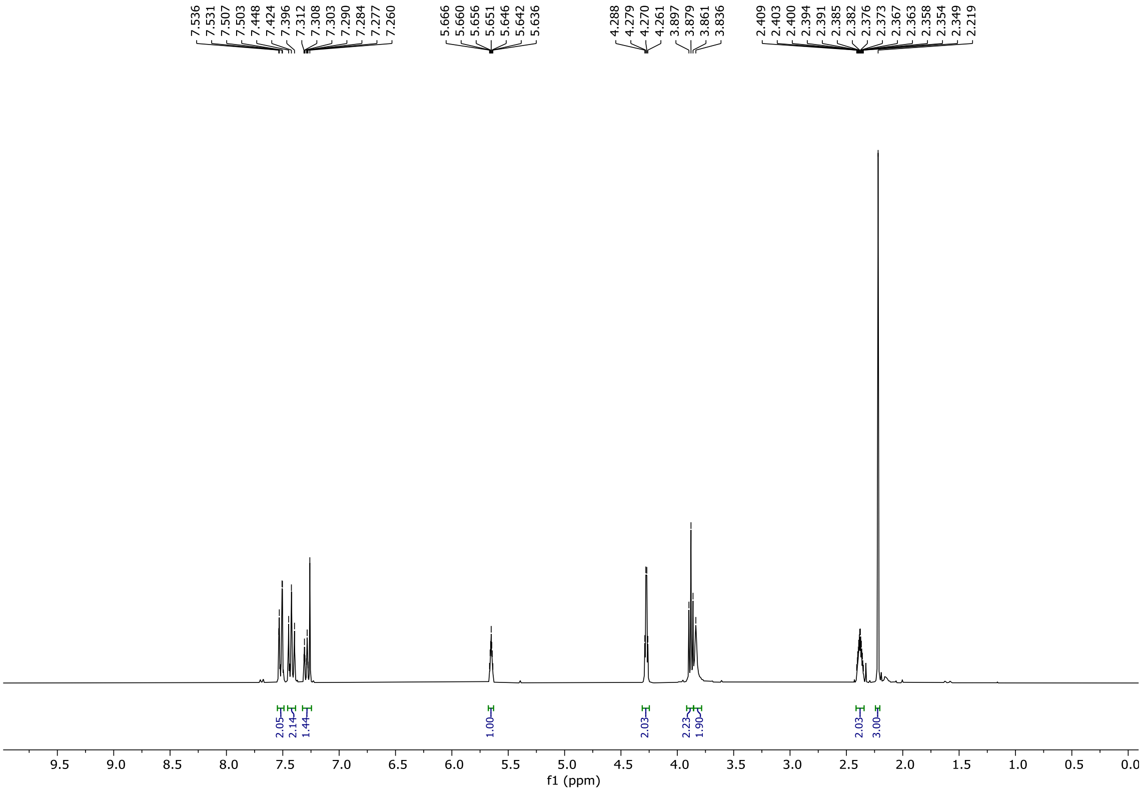

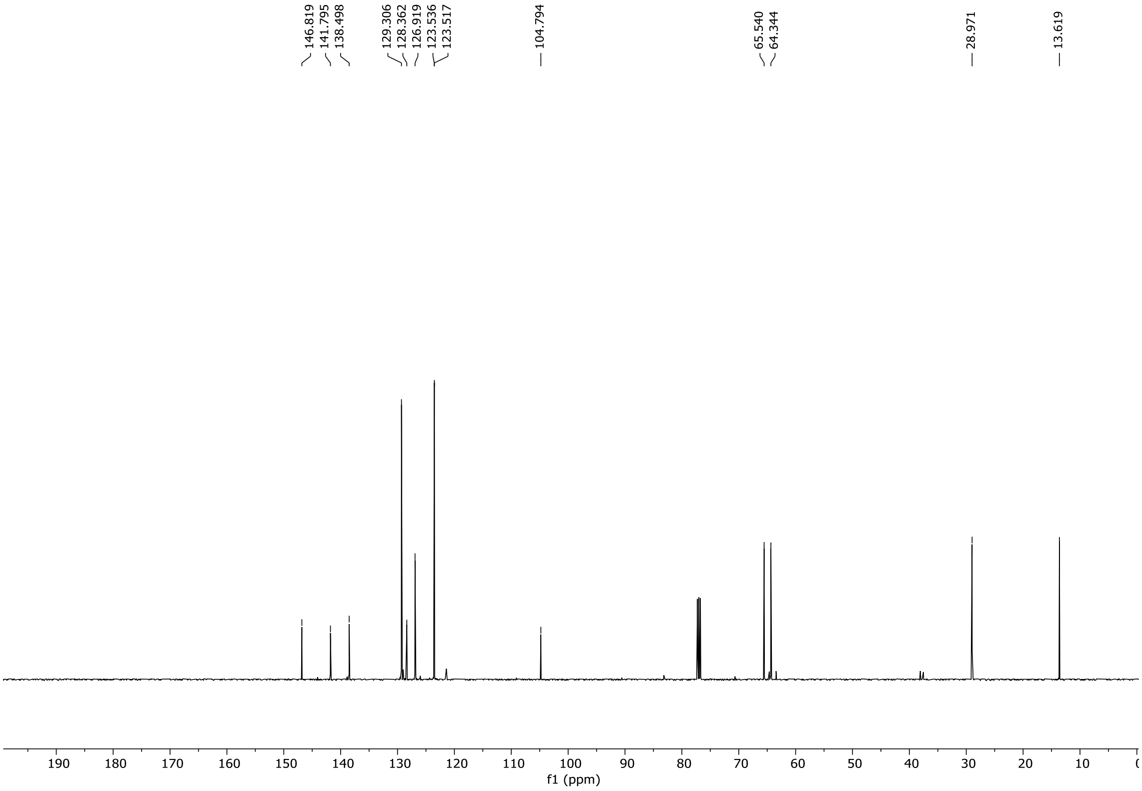

•

Compound **3ea**:

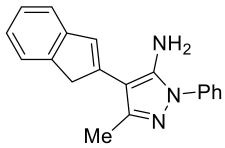

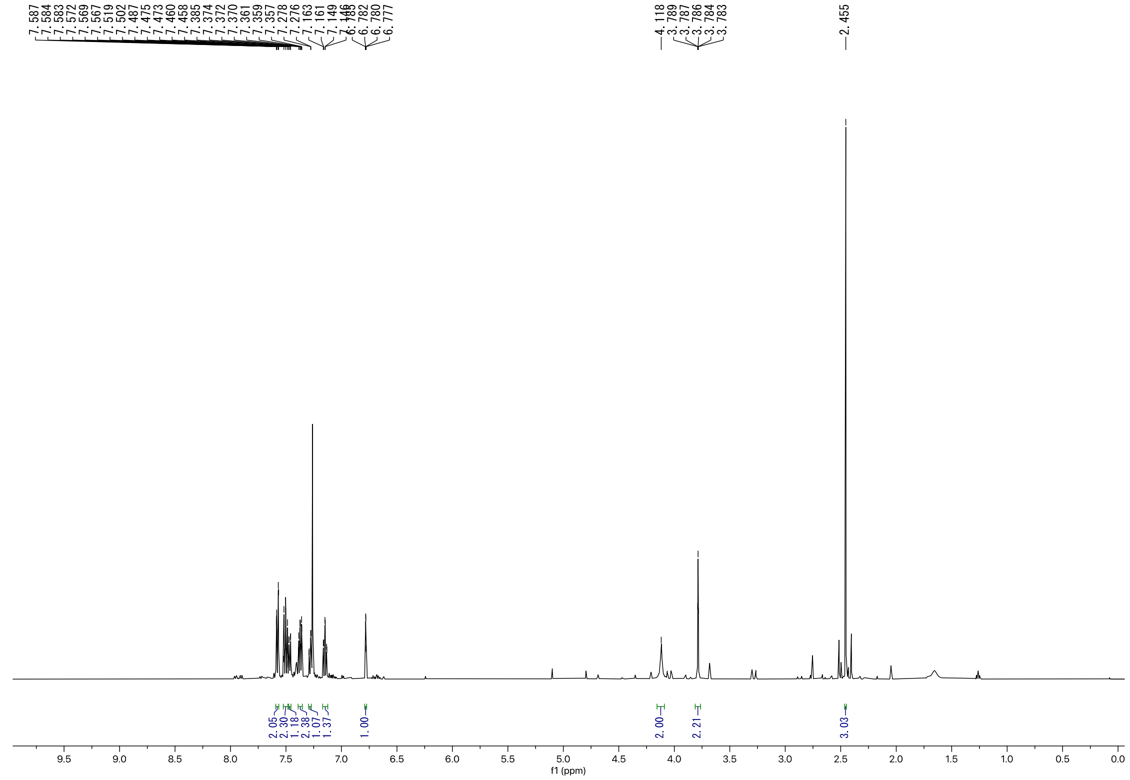

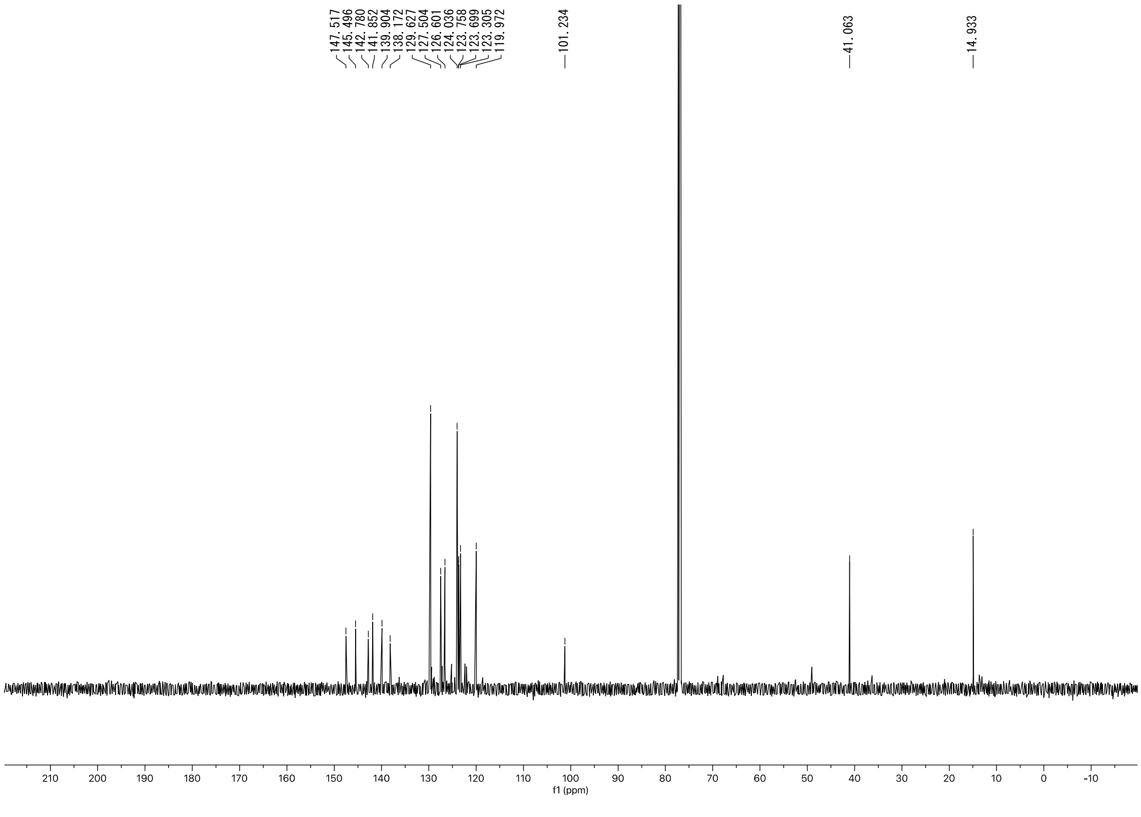

•

Compound **3fa**:

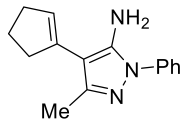

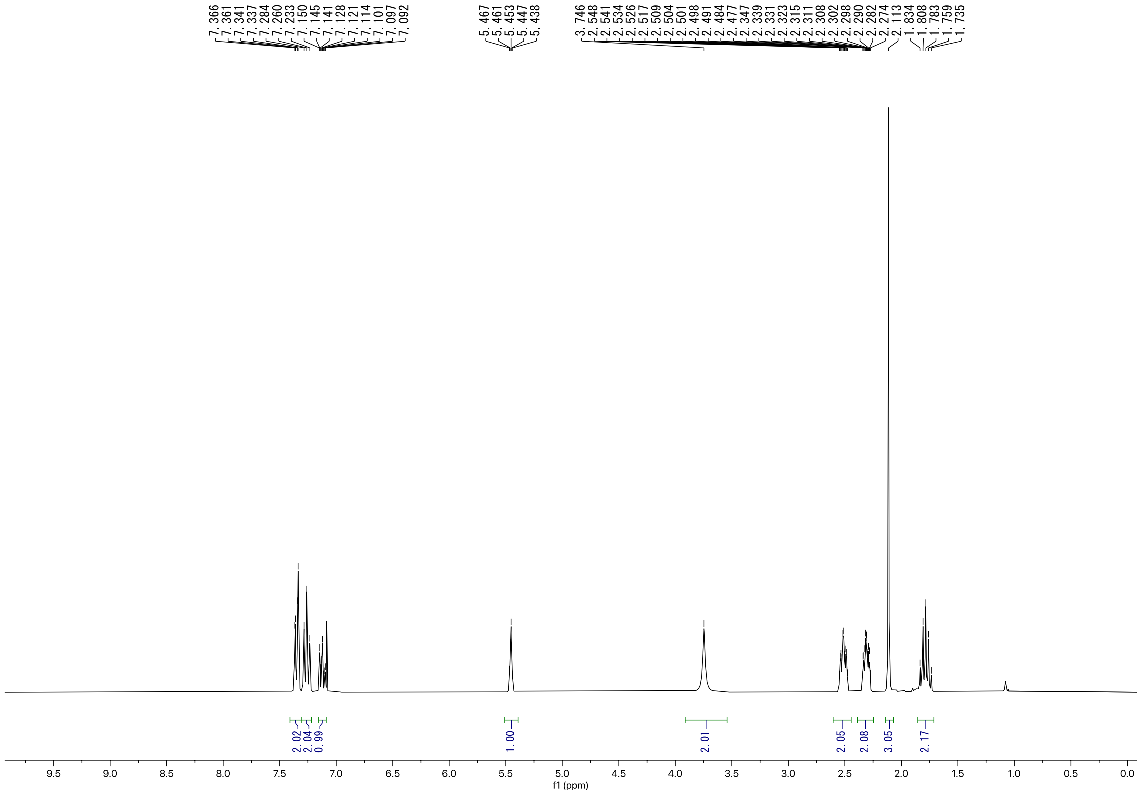

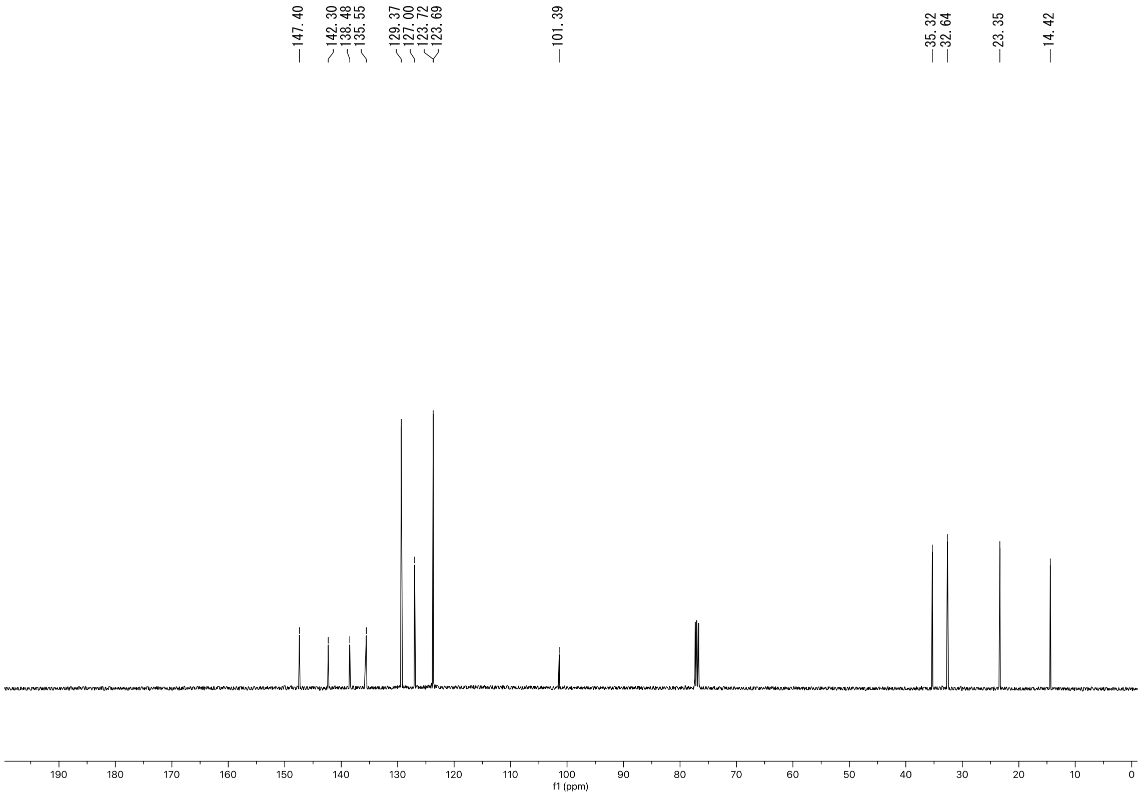

· Compound **3ga**:

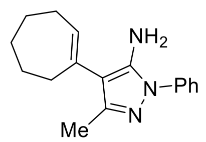

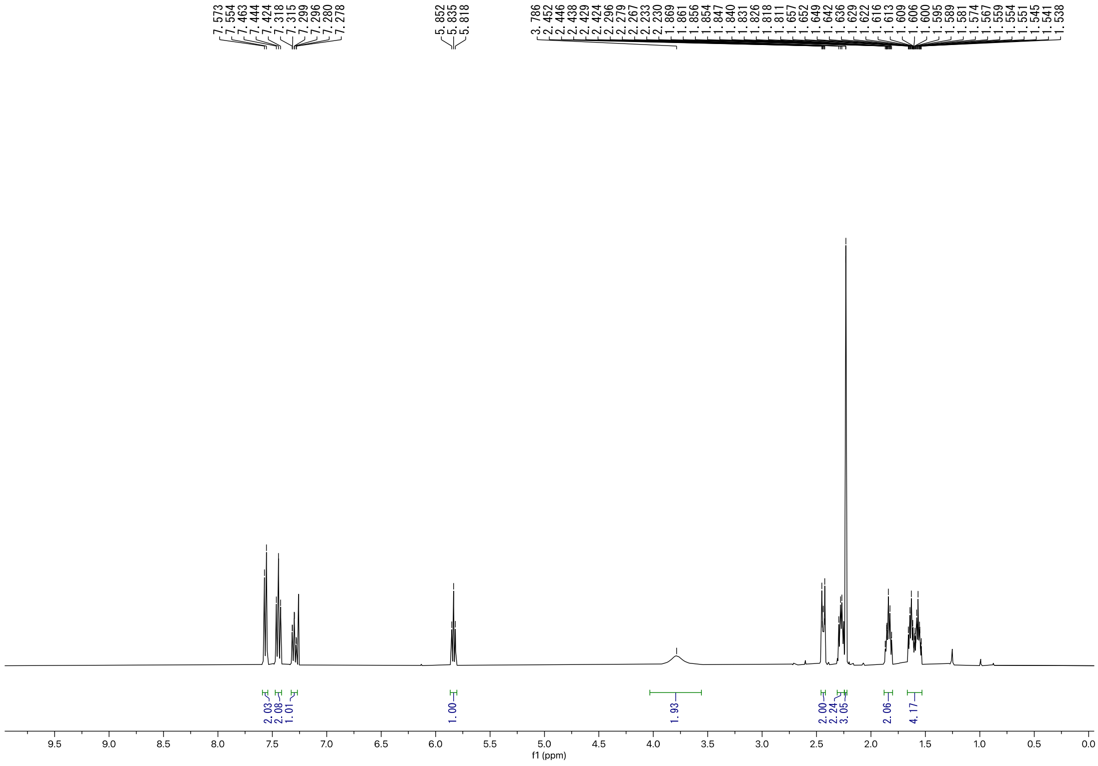

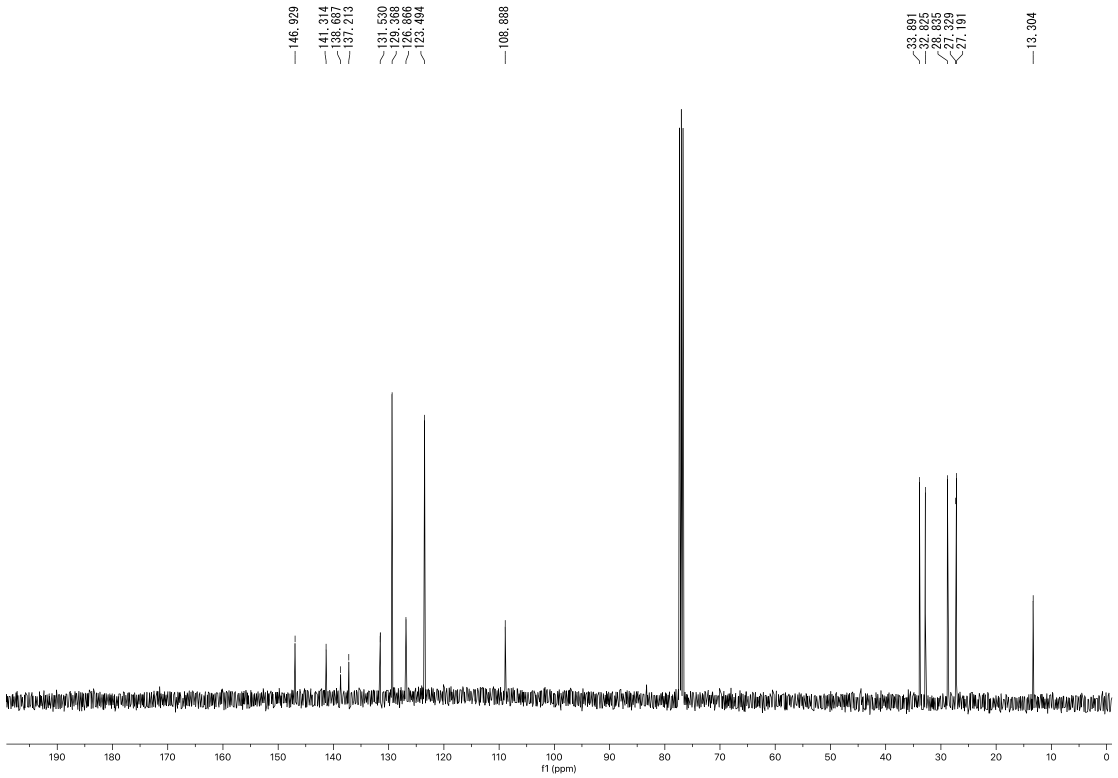

•

Compound **3ab**:

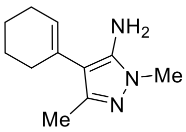

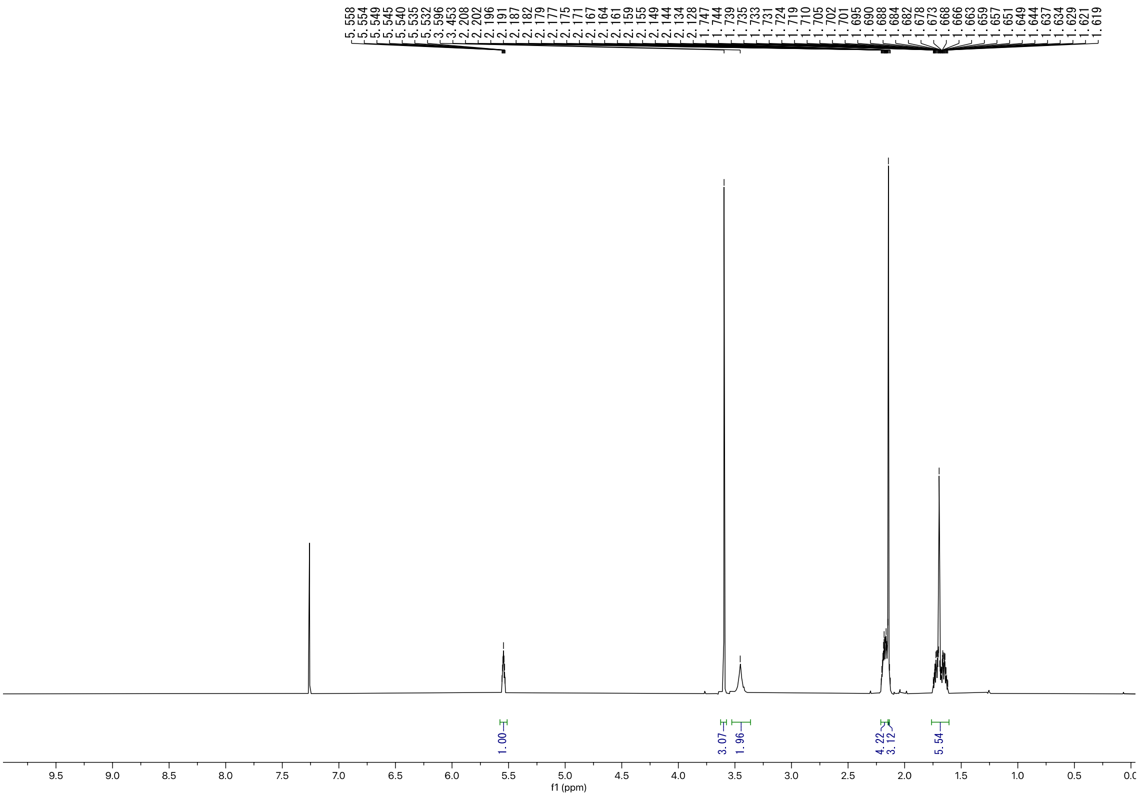

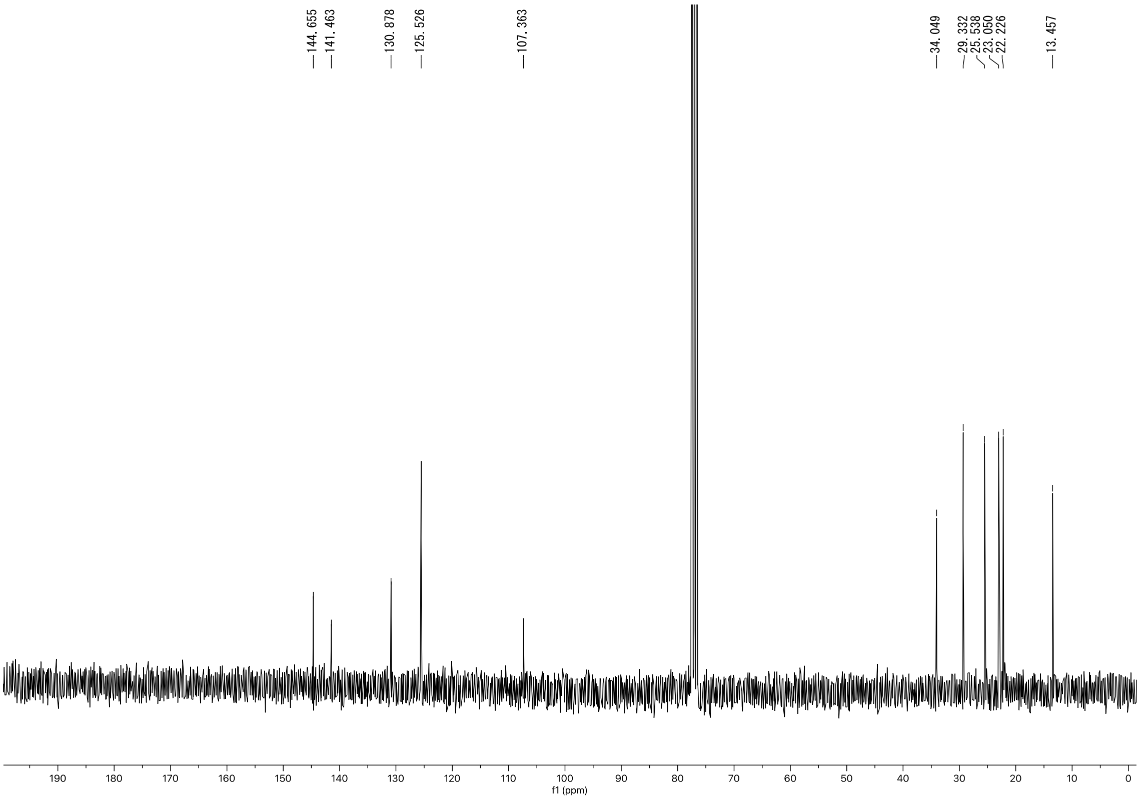

Compound **3ac**:

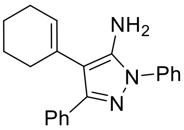

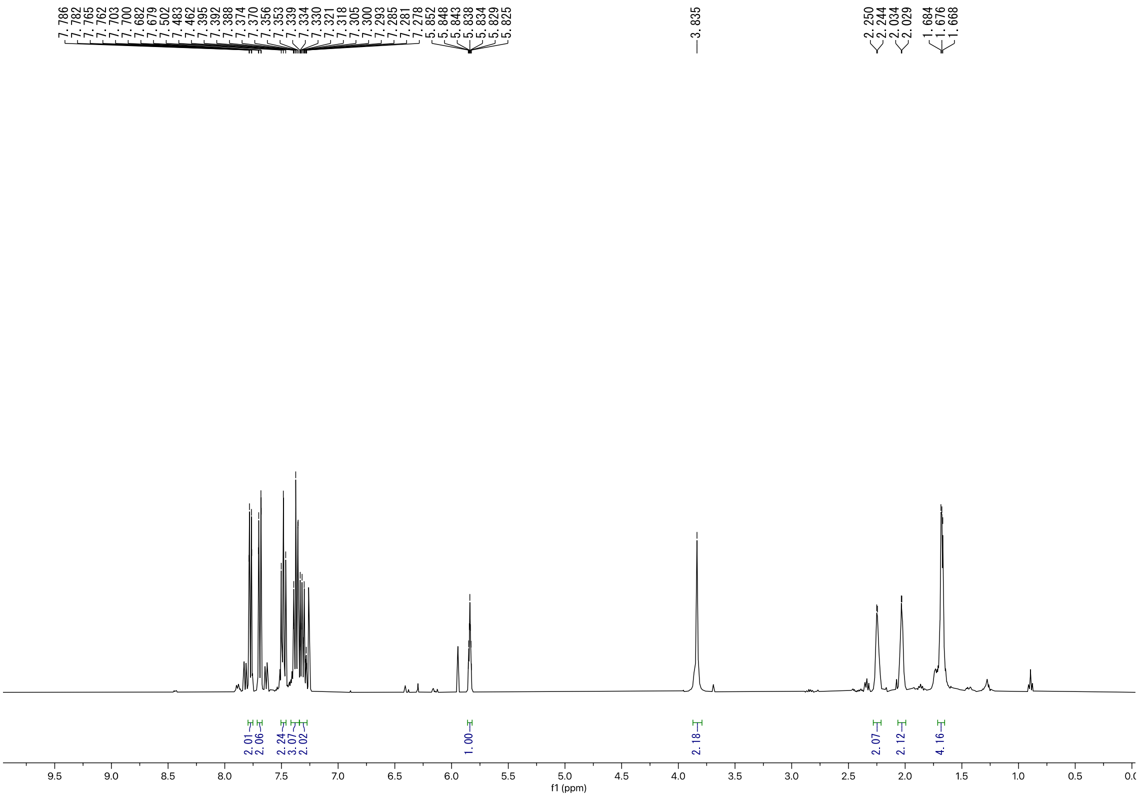

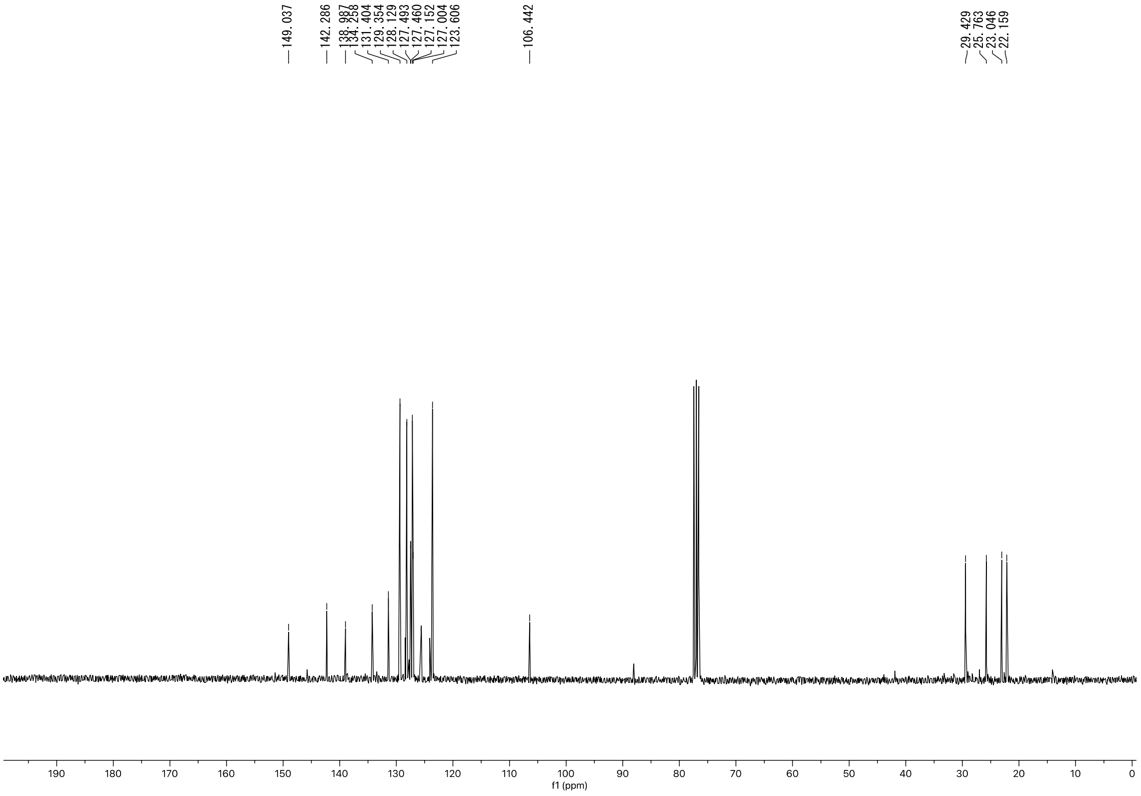

•

Compound **3ad**:

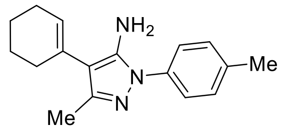

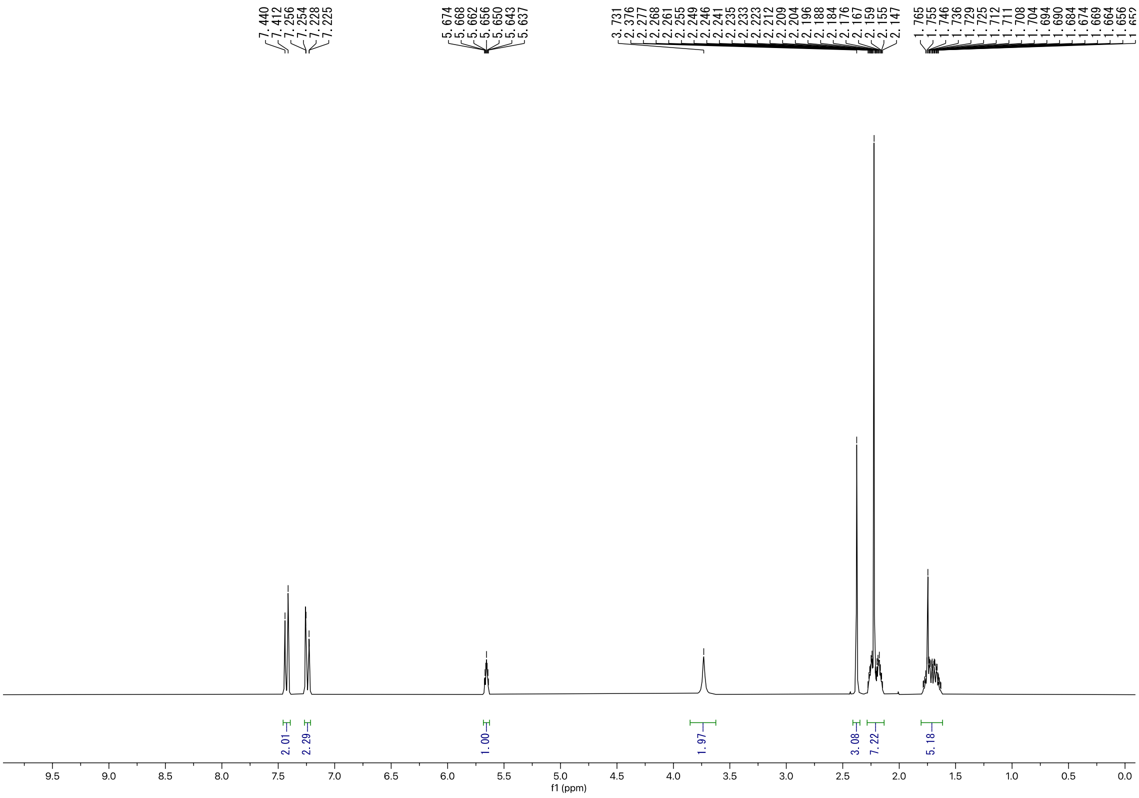

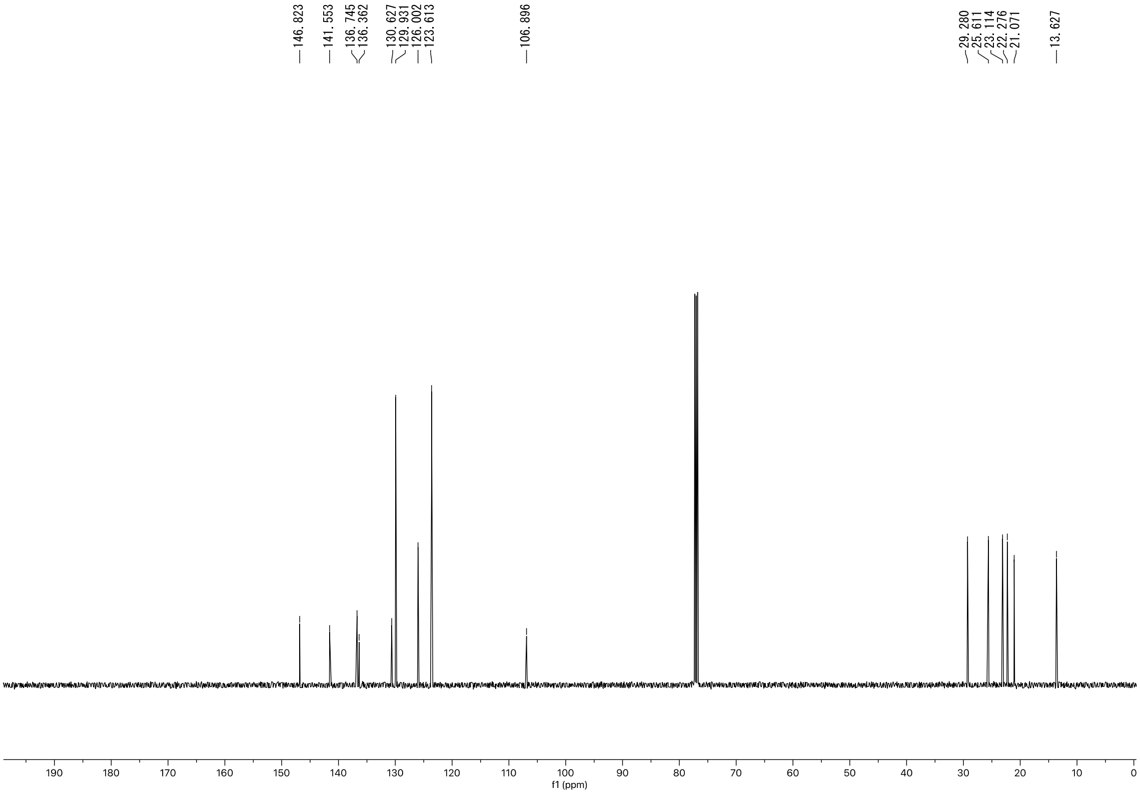

•

Compound **5aaa**:

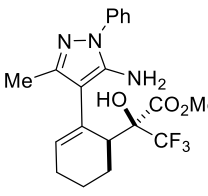

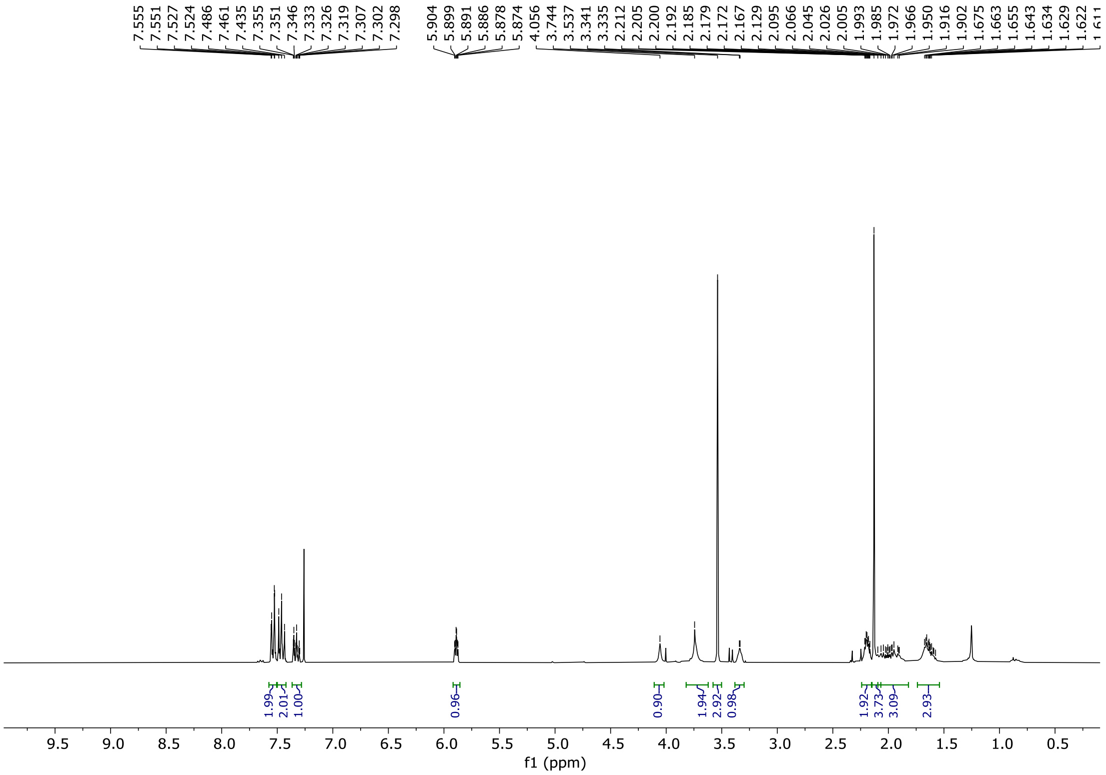

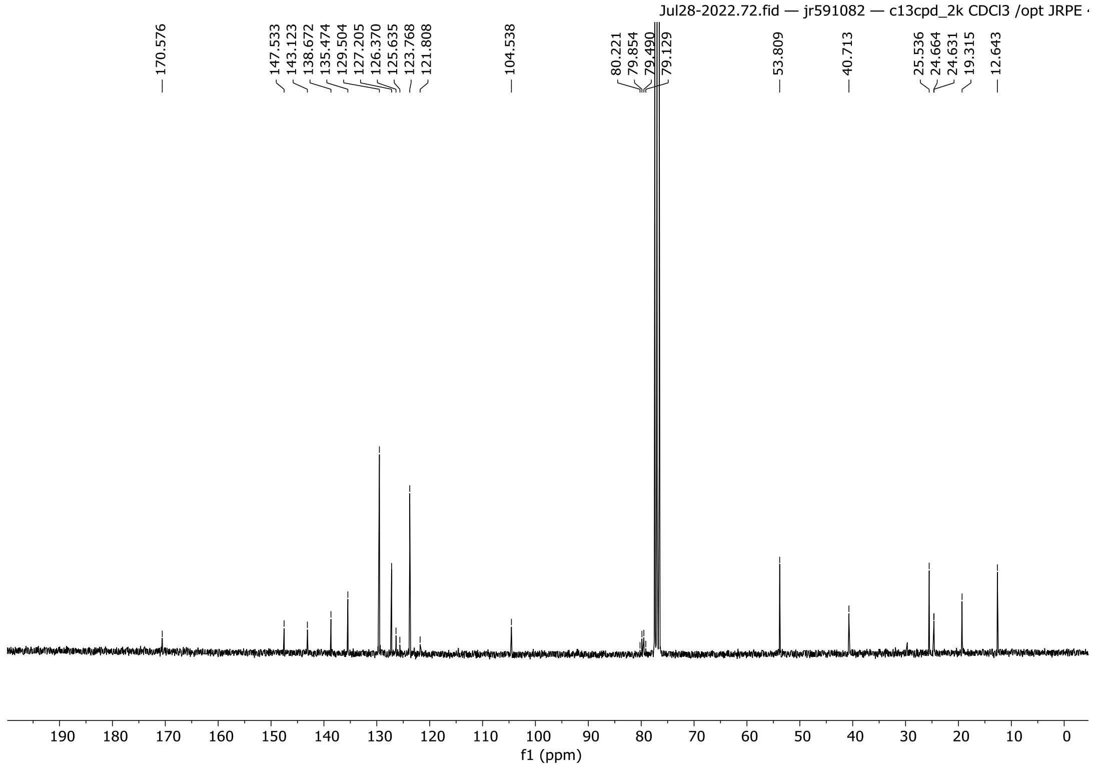

Compound **5aab**:

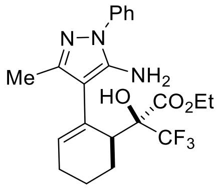

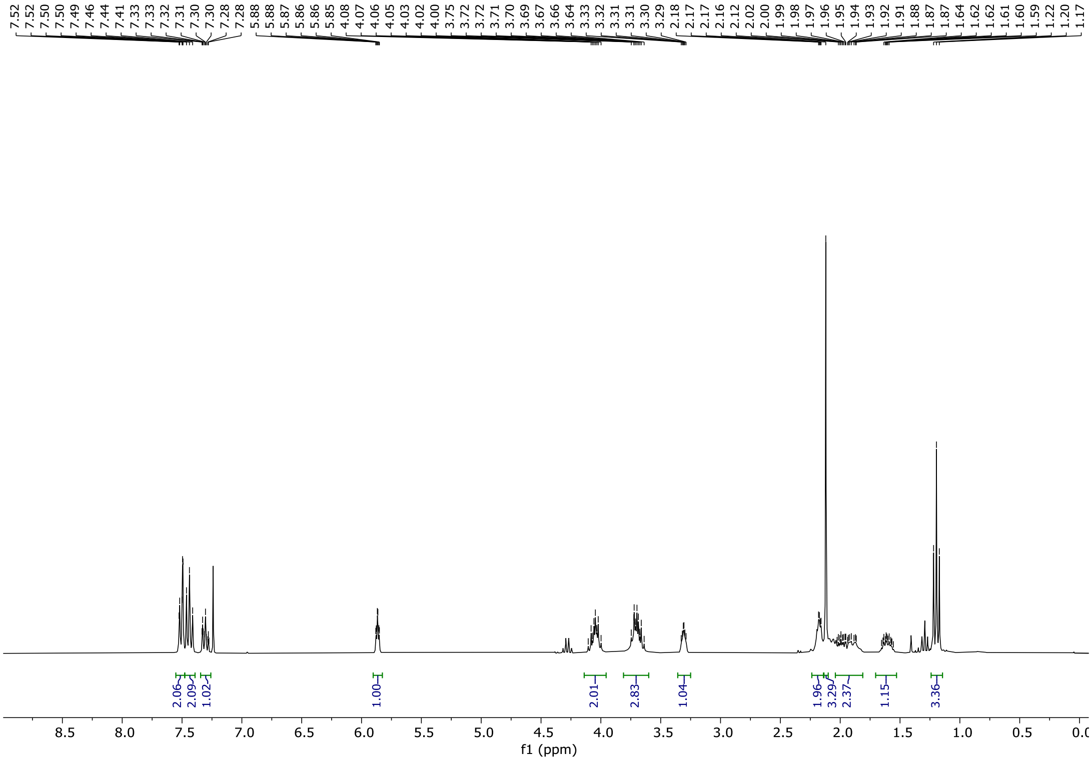

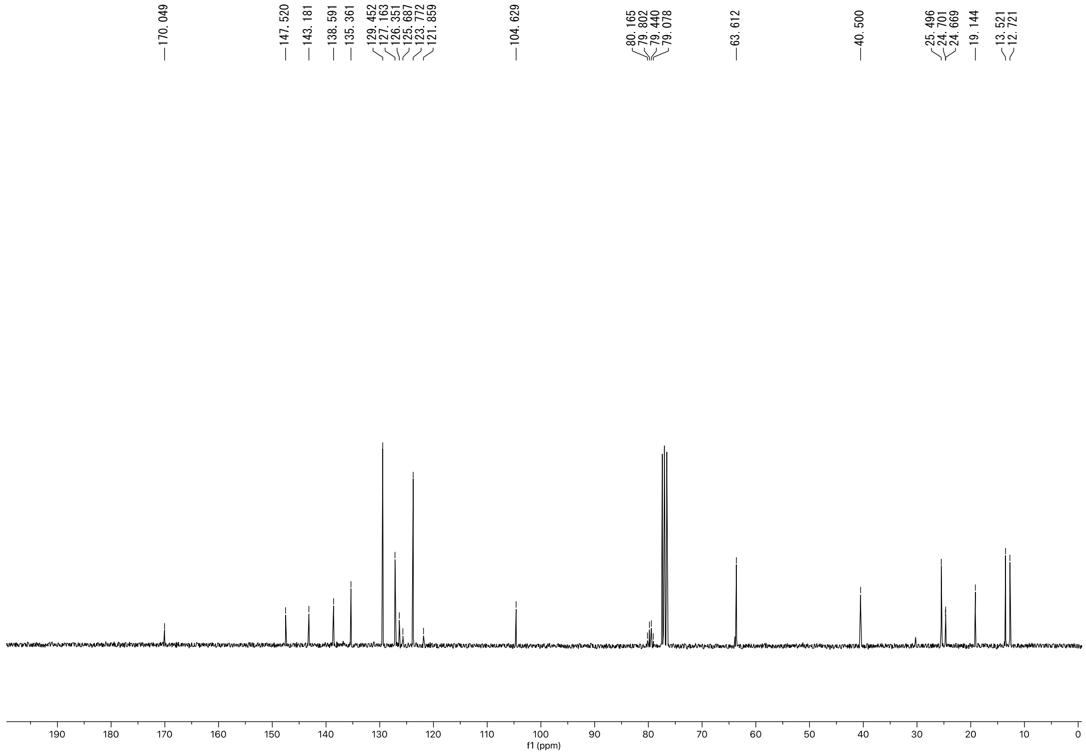

•

Compound **5baa**:

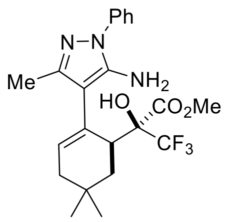

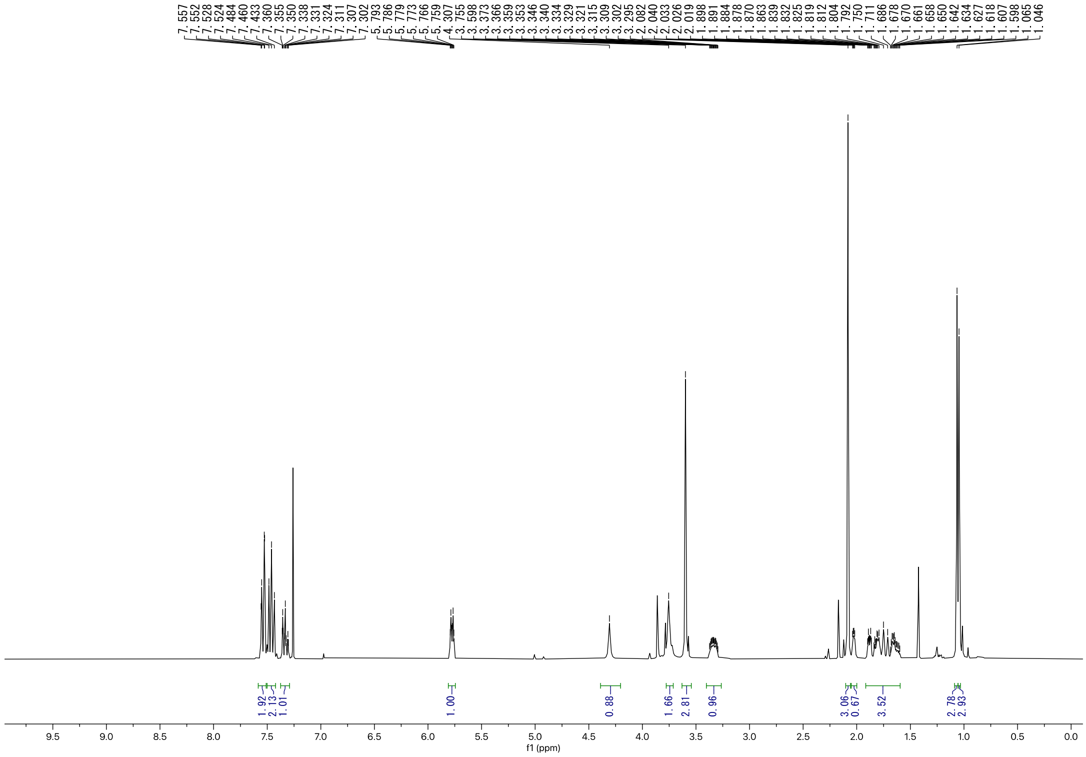

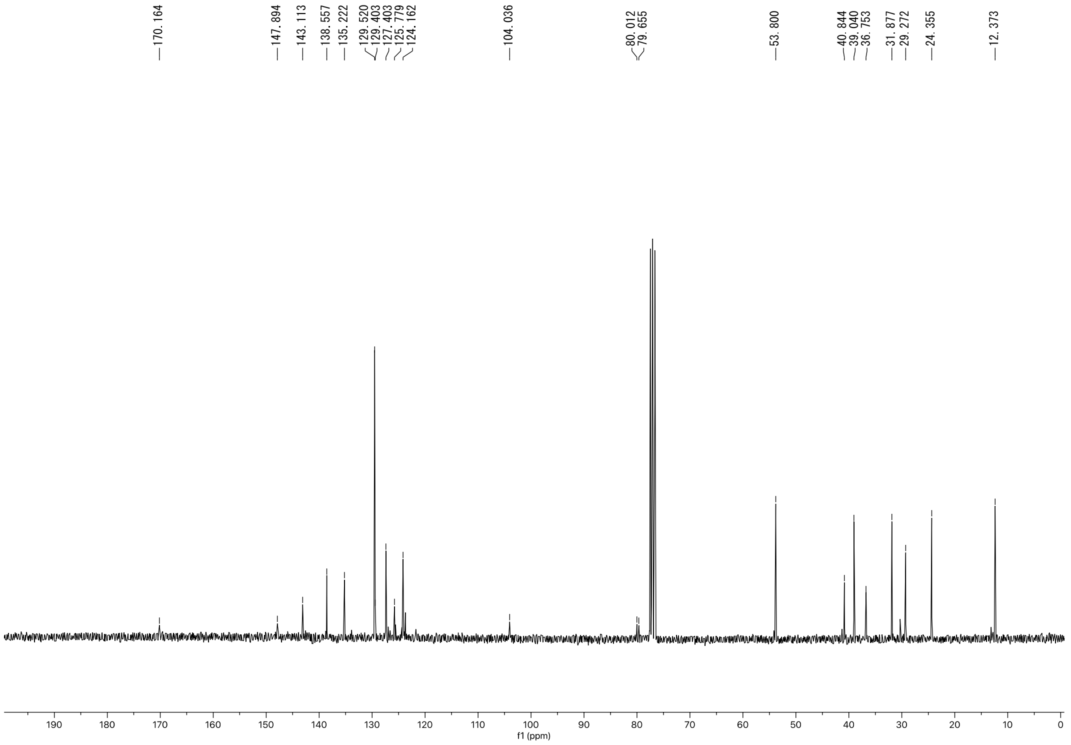

· Compound **5caa**:

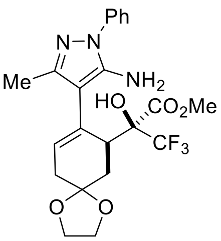

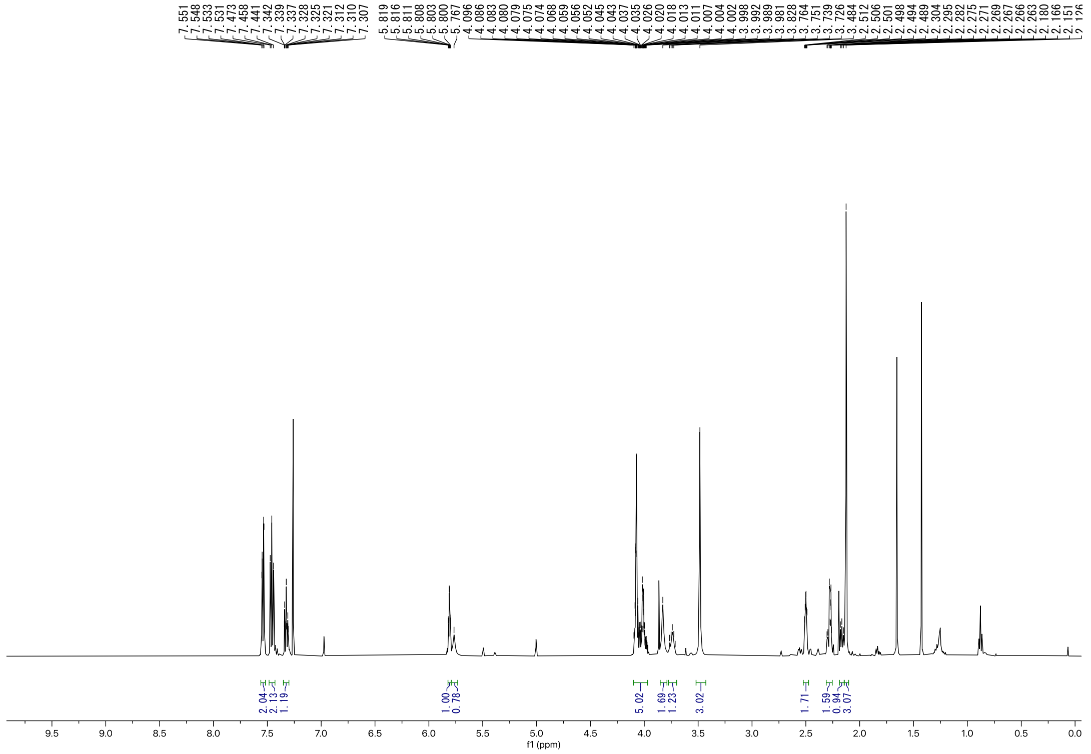

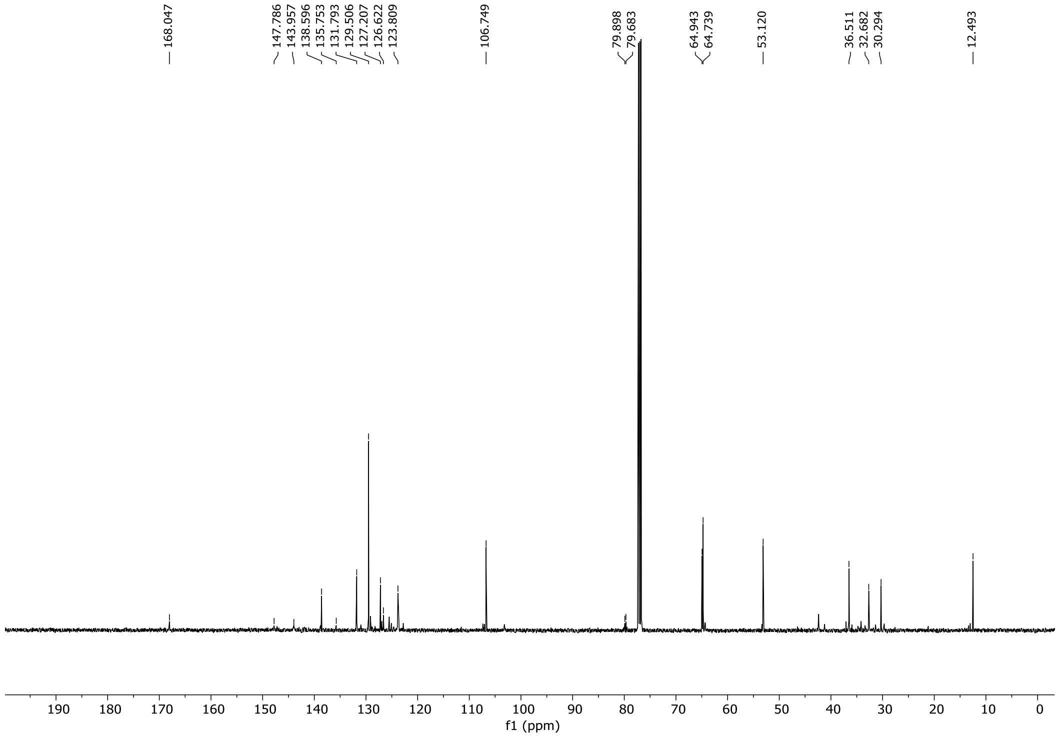

•

Compound **5daa-diastereoisomer 1**:

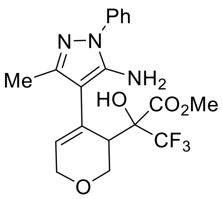

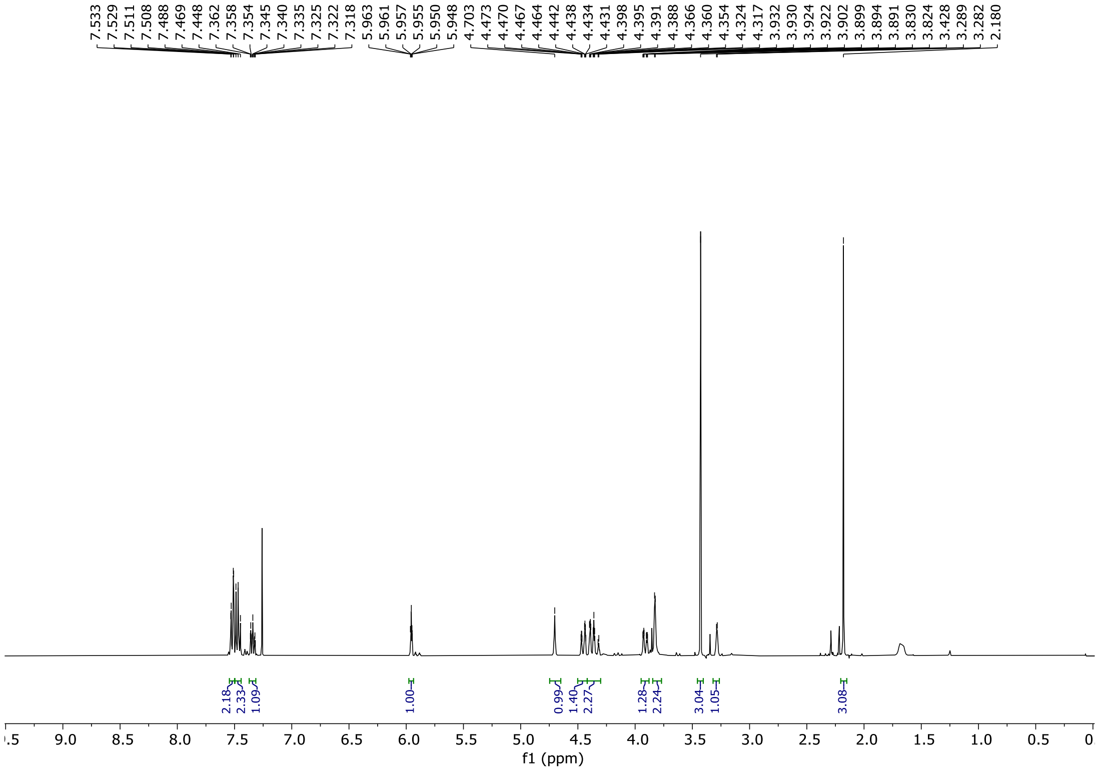

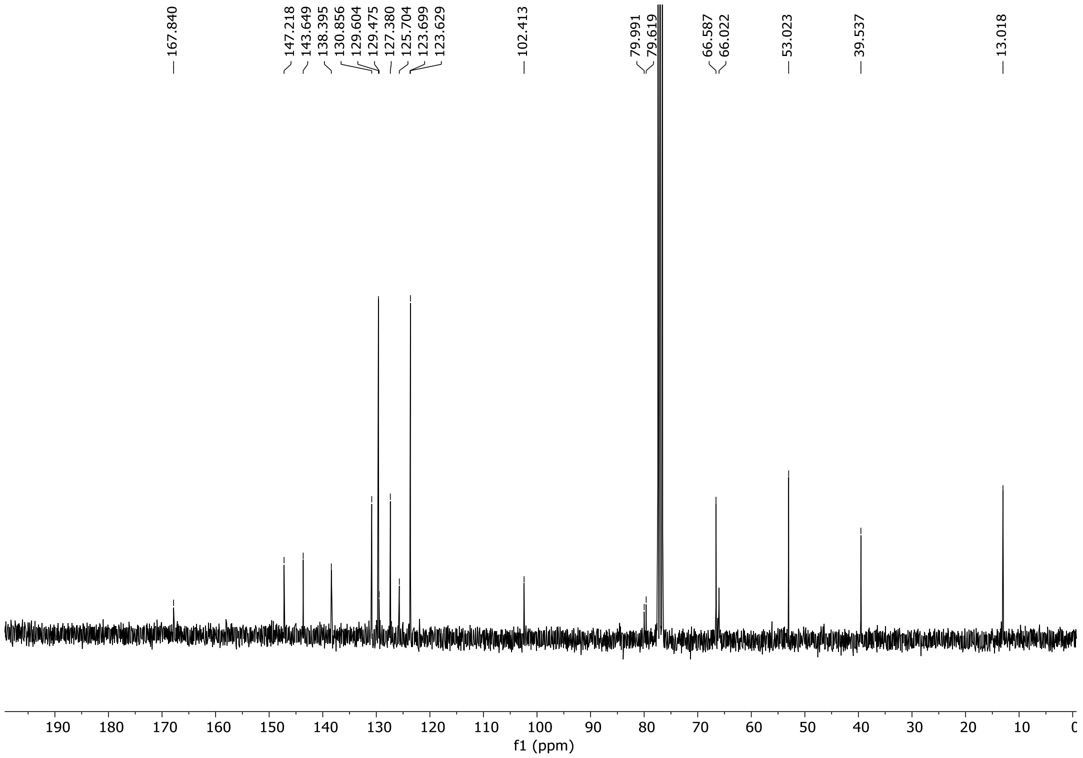

•

Compound **5daa-diastereoisomer 2**:

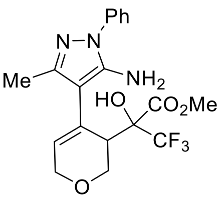

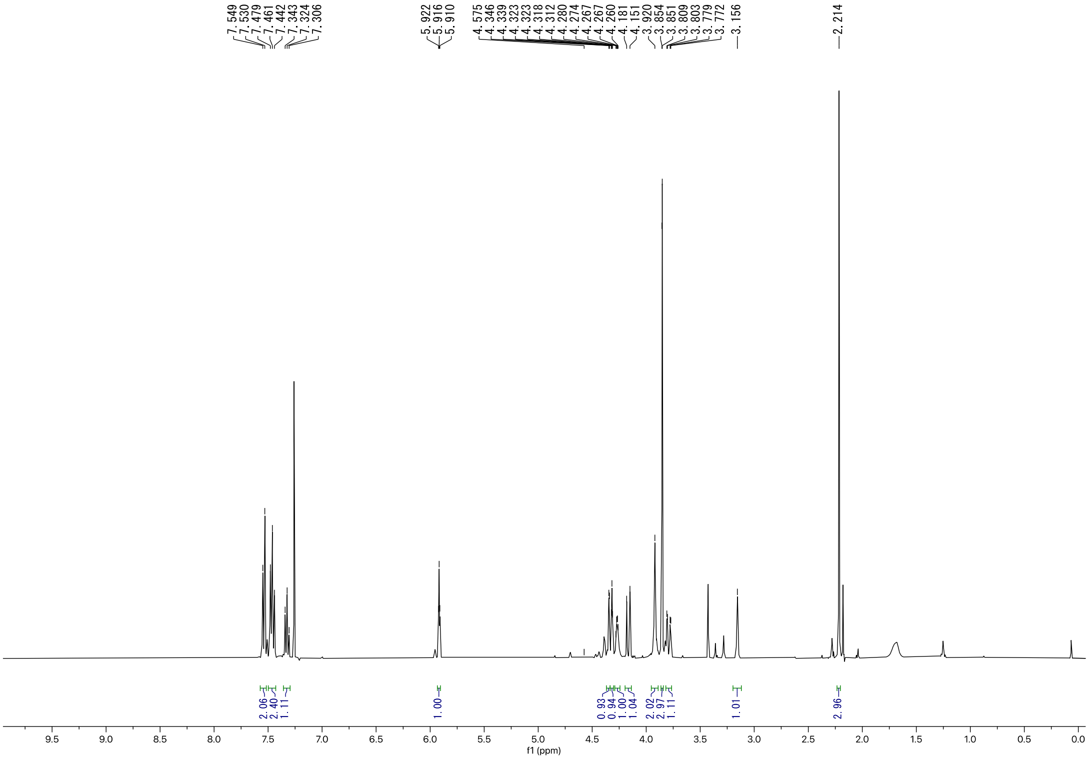

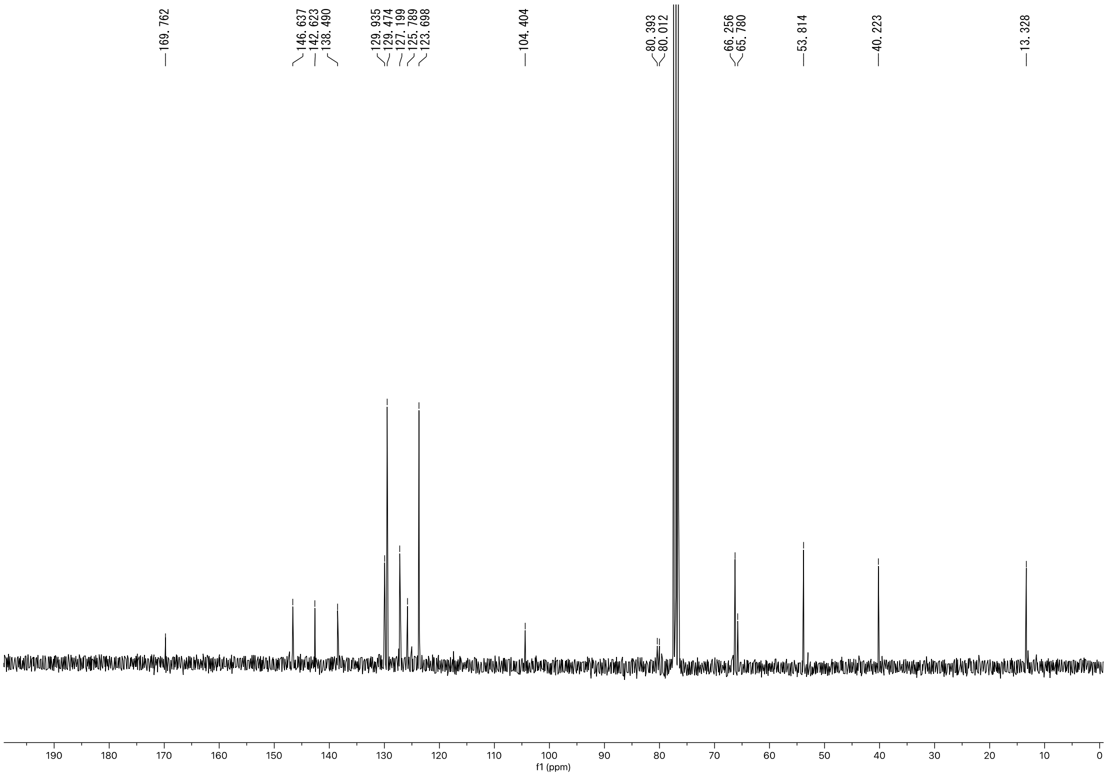

.

Compound **5faa**:

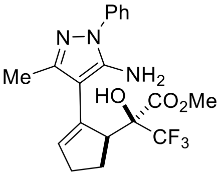

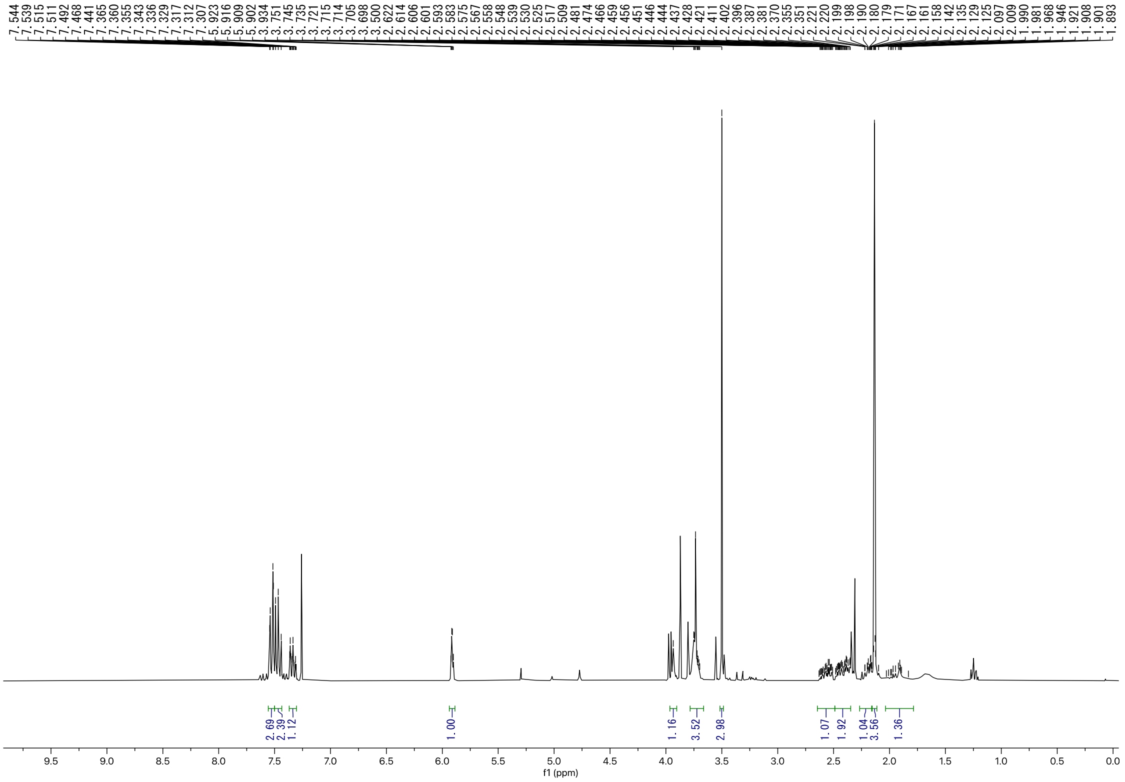

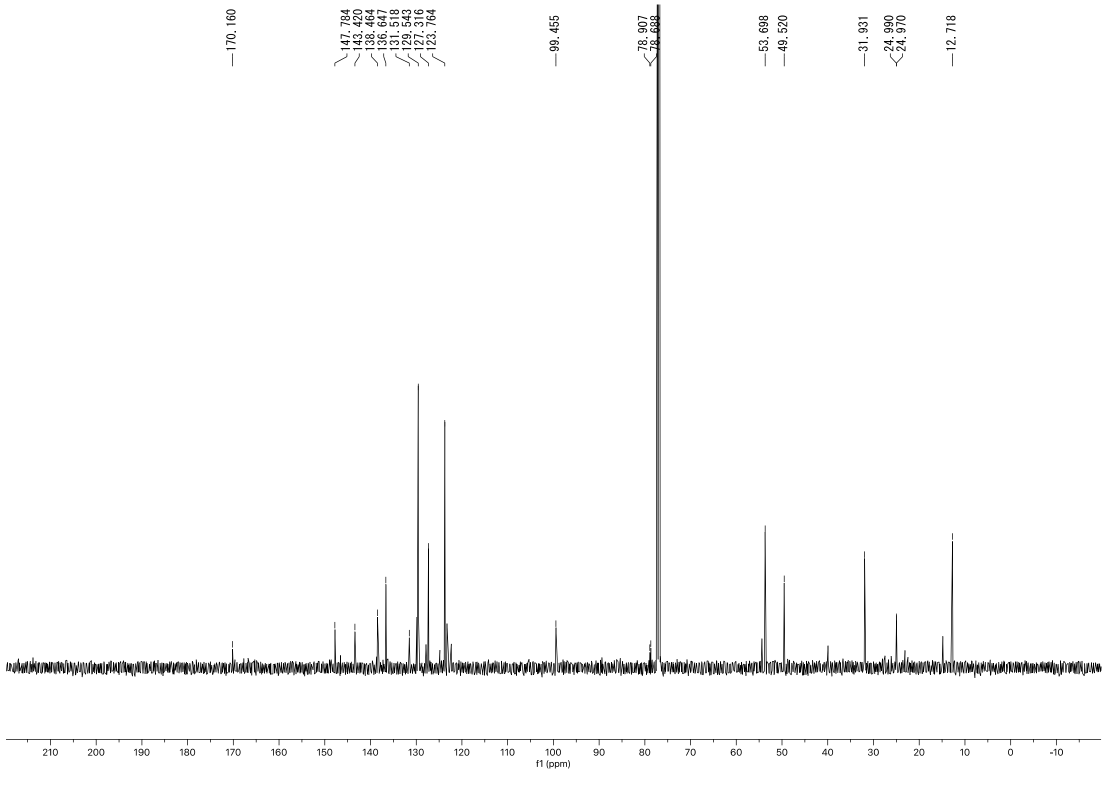

.

Compound **5gaa**:

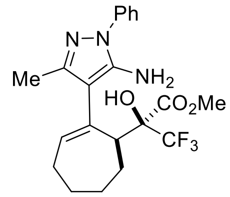

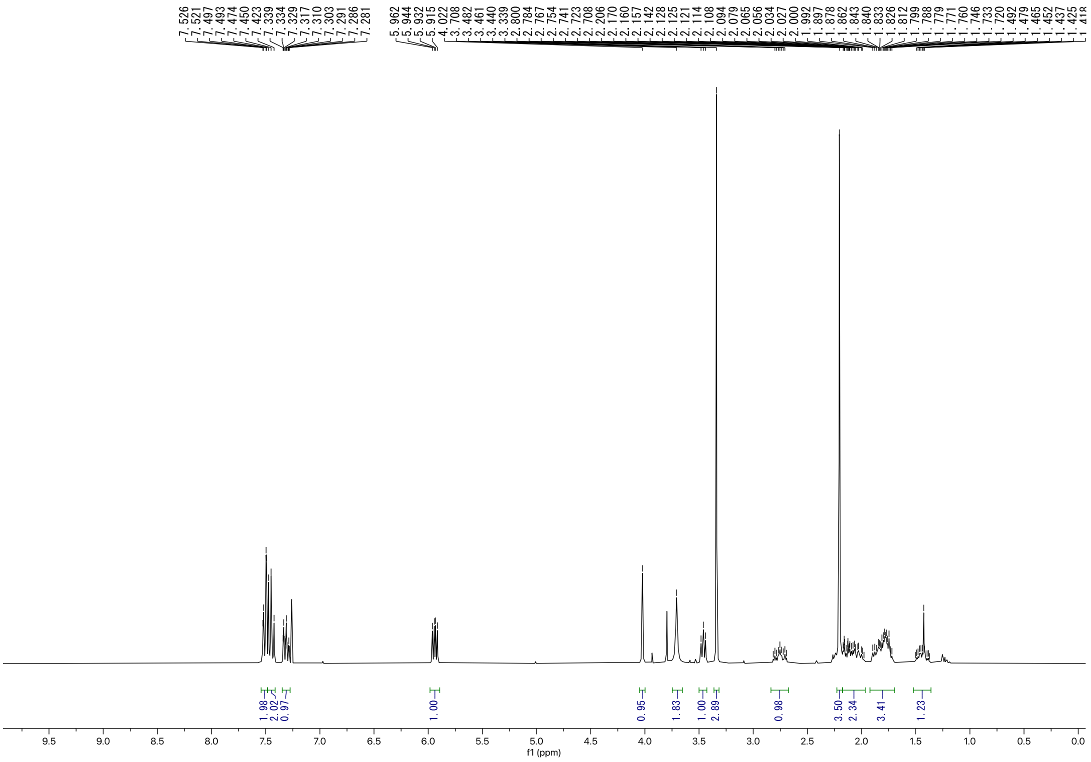

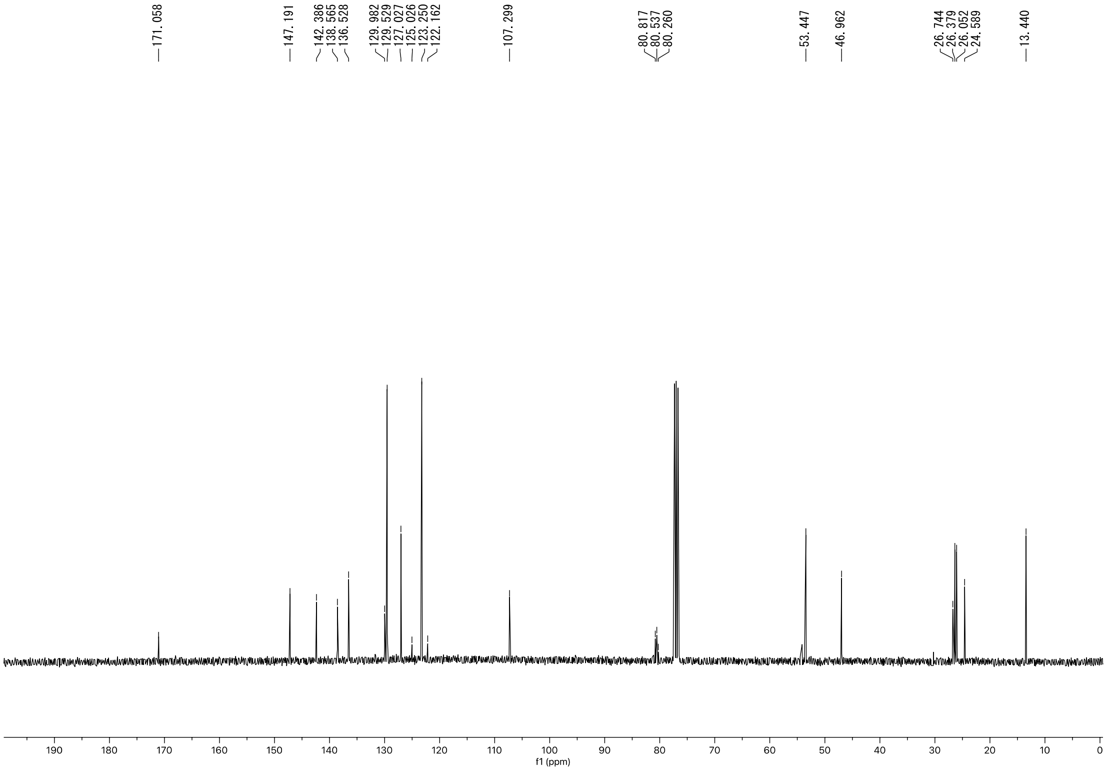

•

Compound **5aba**:

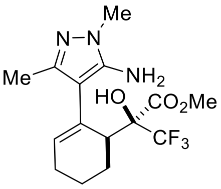

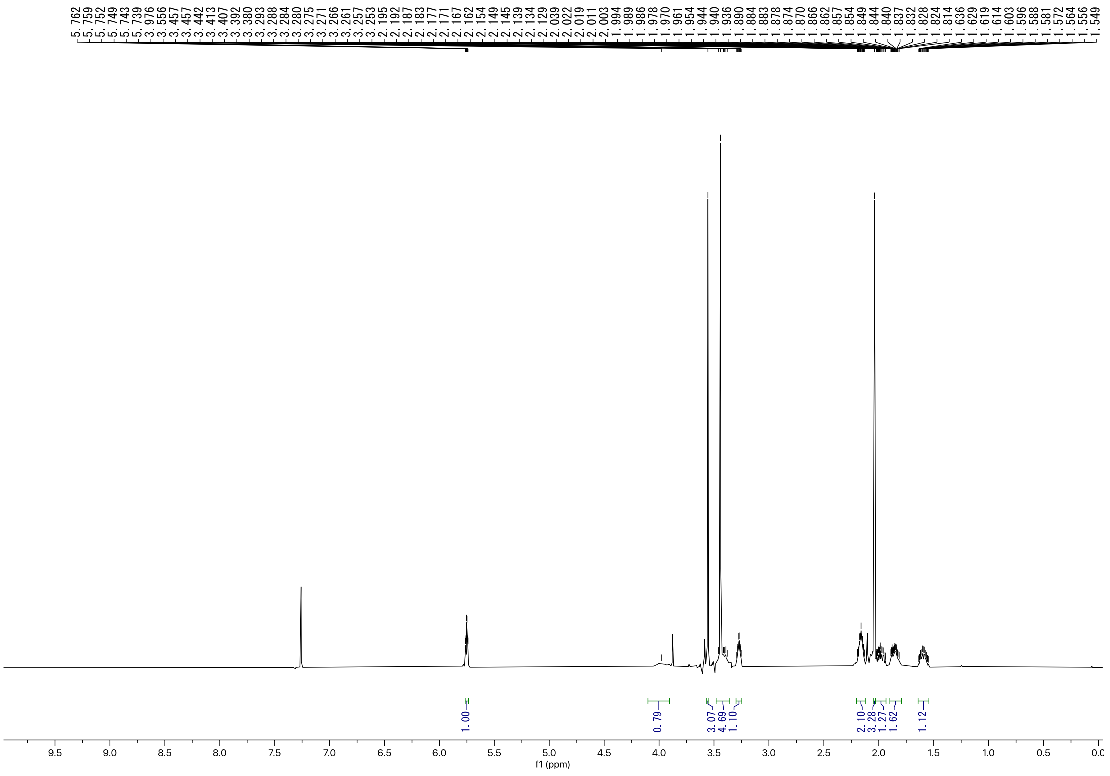

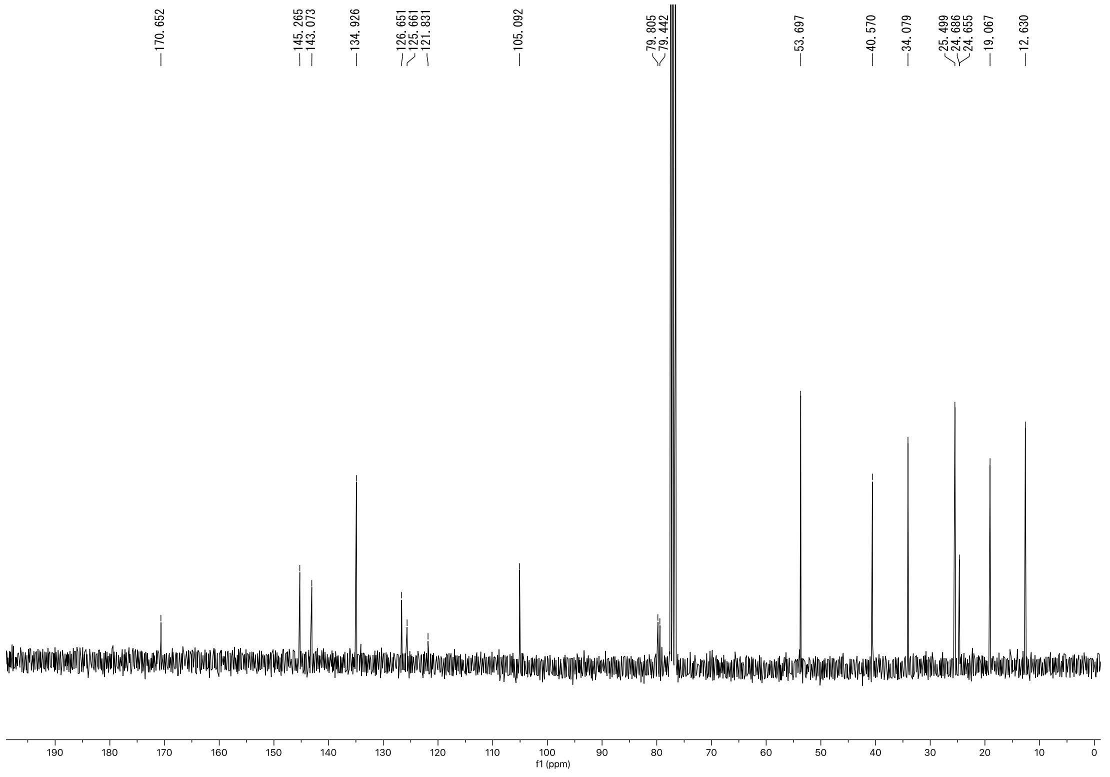

•

Compound **5aca**:

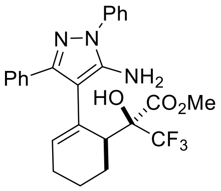

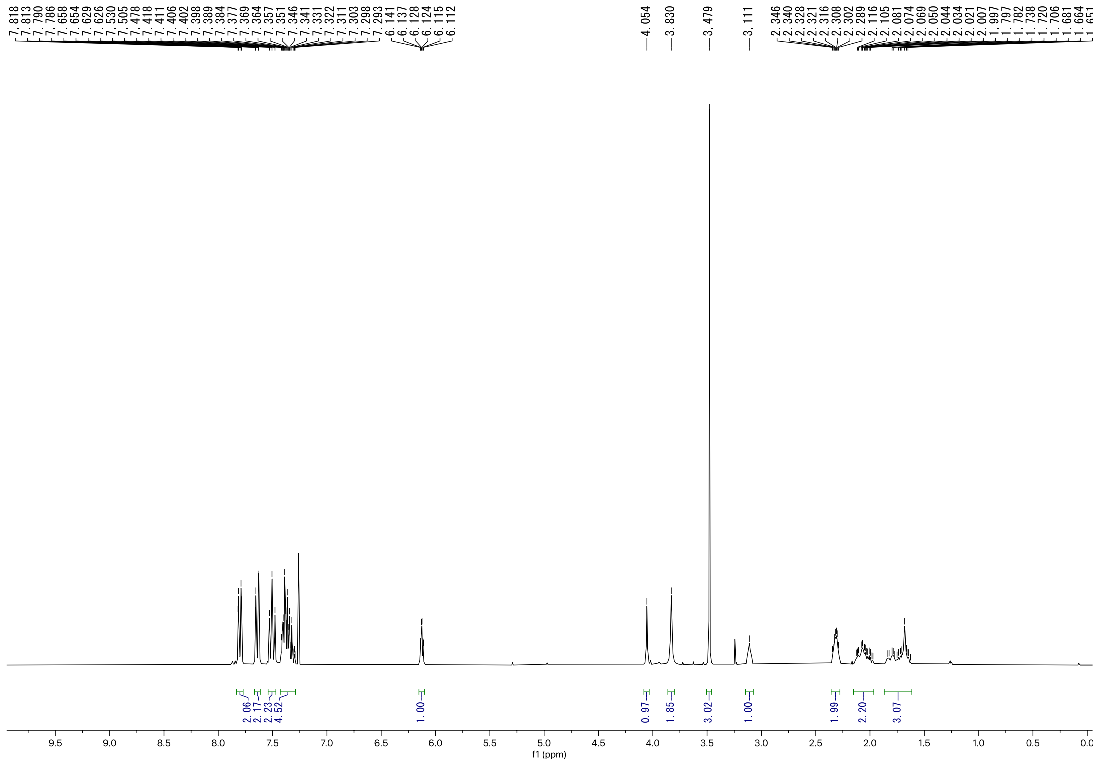

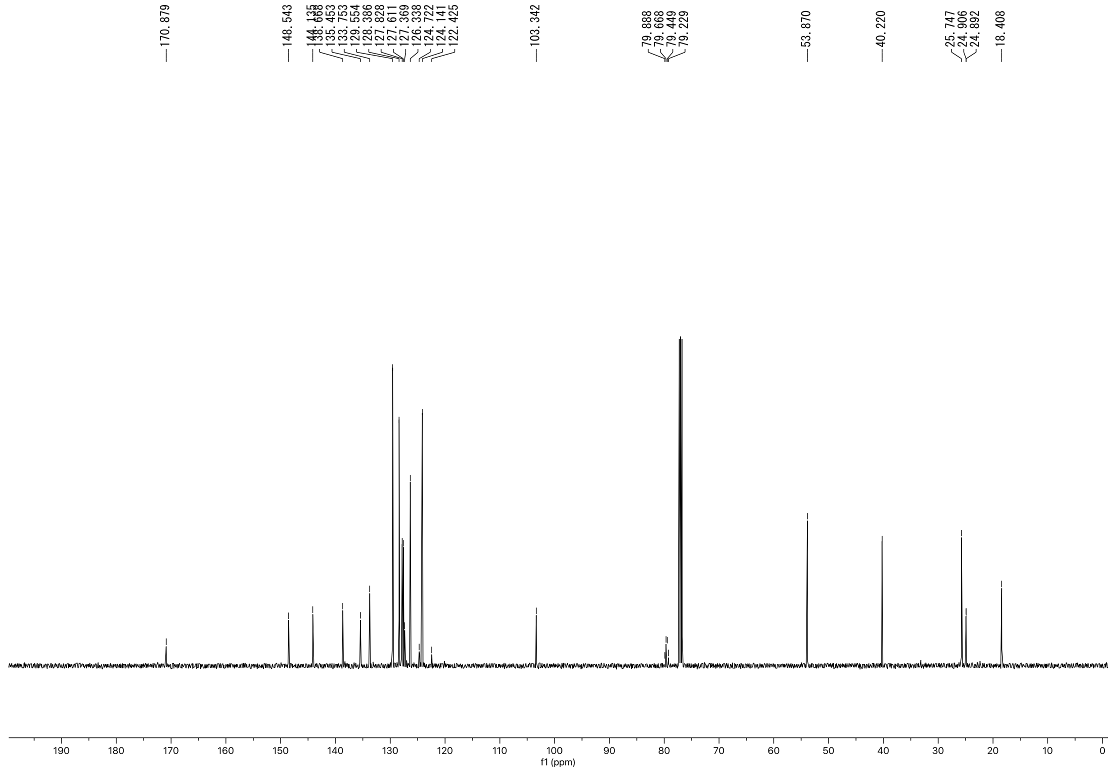

•

Compound **5ada**:

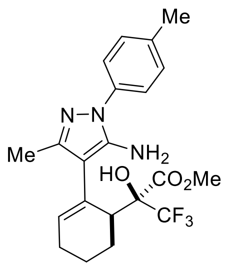

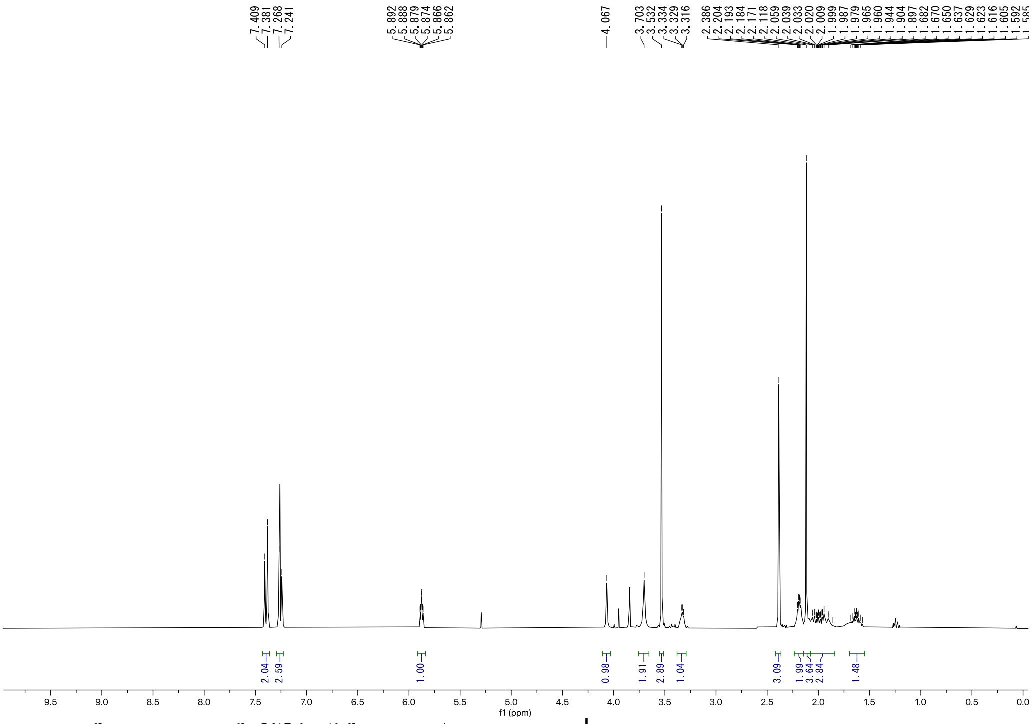

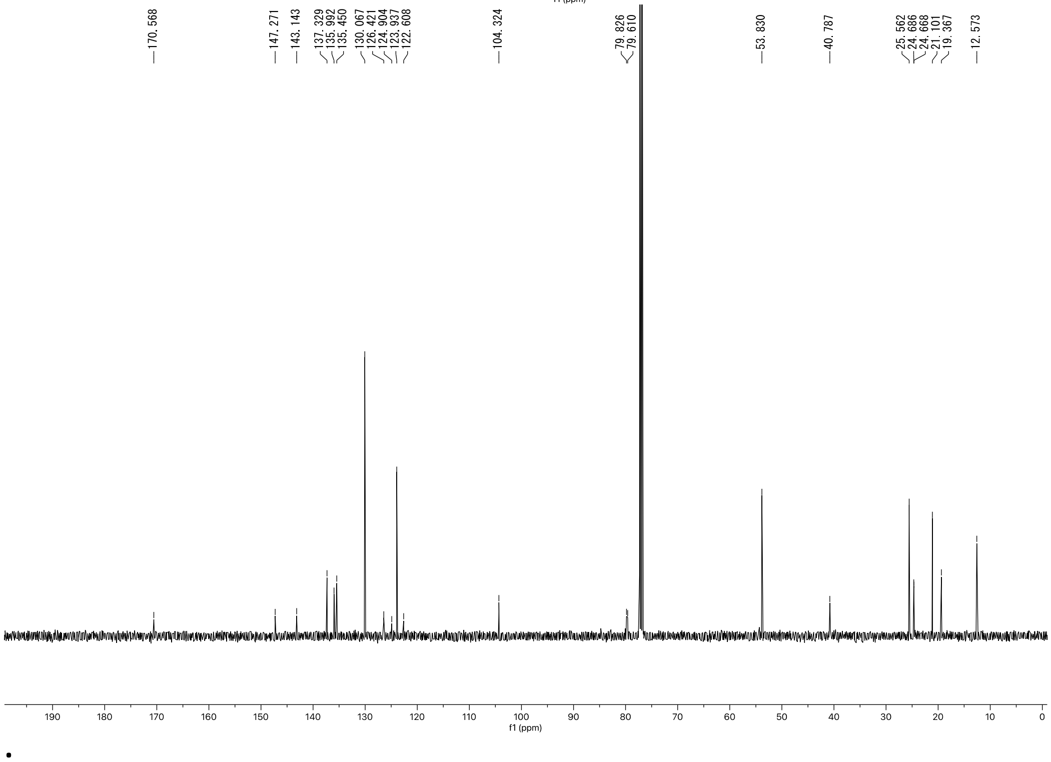

•

Compound **7**:

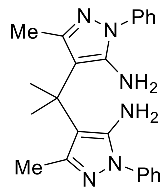
Me
N
N
Ph
NH2
NH2
Me
N
N
Ph

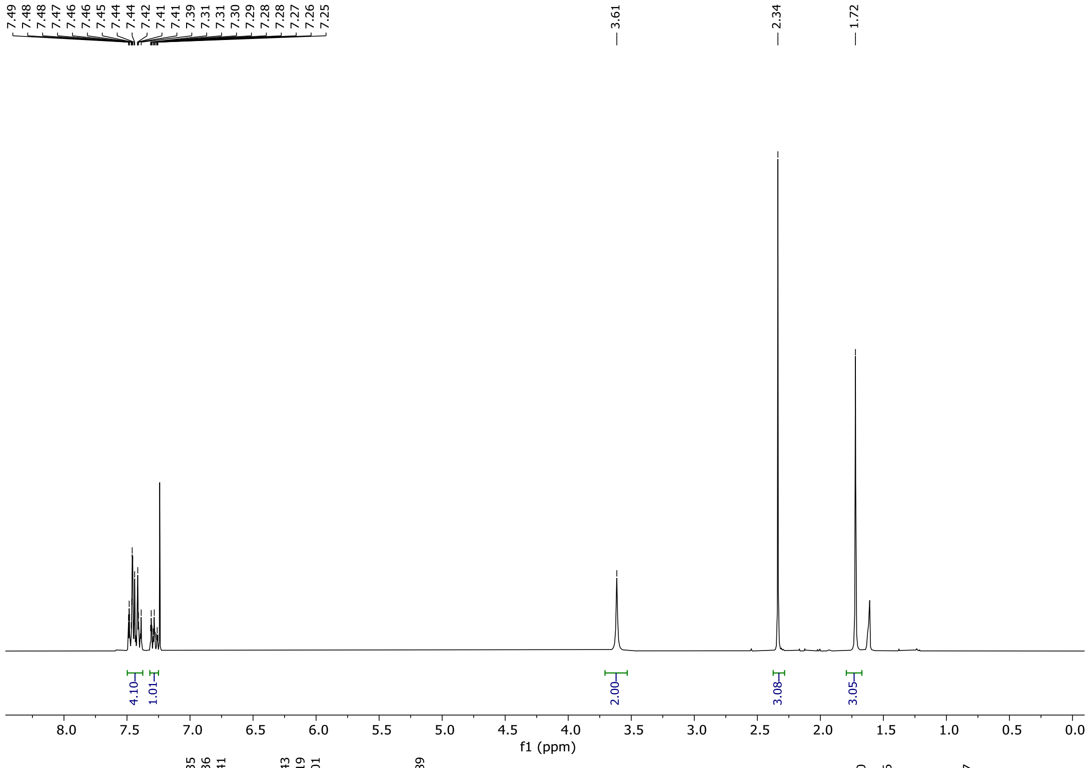
7.49
7.48
7.48
7.47
7.46
7.46
7.45
7.44
7.44
7.42
7.41
7.41
7.39
7.31
7.31
7.30
7.29
7.28
7.28
7.27
7.26
7.25
3.61
2.34
1.72
4.10
1.01
2.00
3.08
3.05
f1 (ppm)

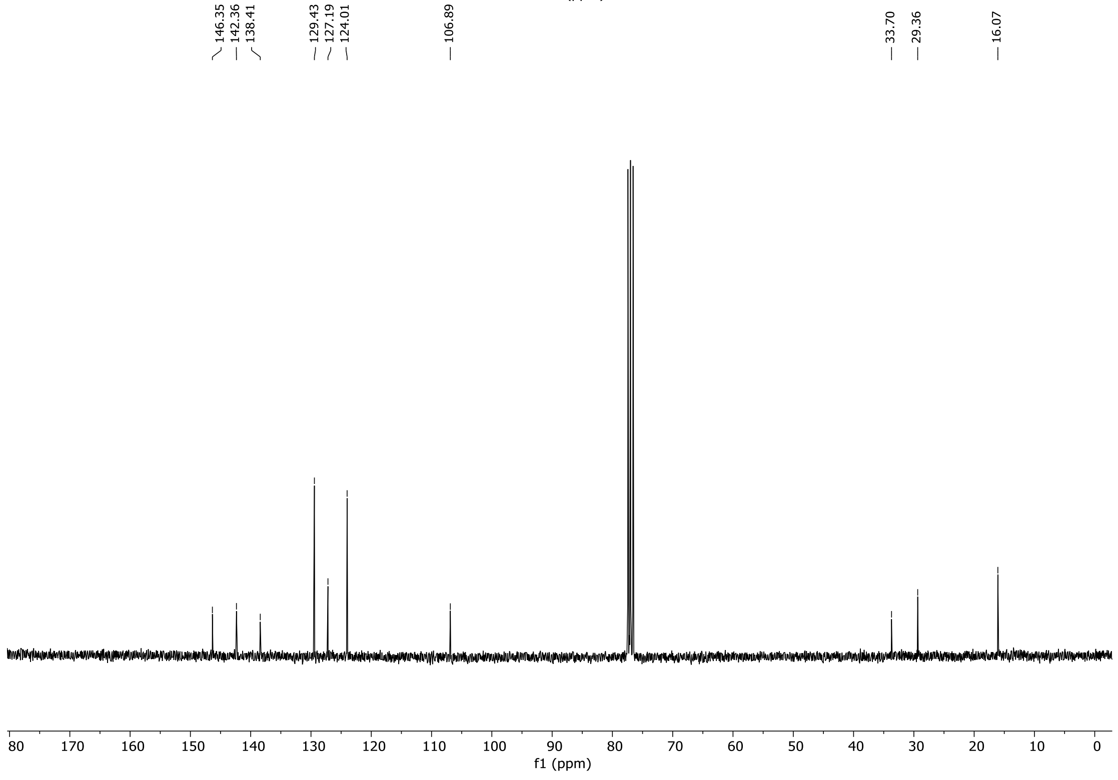
146.35
142.36
138.41
129.43
127.19
124.01
106.89
33.70
29.36
16.07
f1 (ppm)

7. HPLC DATA

· Compound **5aaa**:

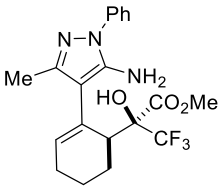

Data Path: C:\WIN32APP\HSM\CARLOS\DATA\LC-1032 AYH 8020 1mL\
Processing Method: Carlos-2

Application: Carlos
Sample Name: LC-1032 AYH 8020 1mL

Series:LC-1032 AYH 8020 1mL
Vial Number: 1

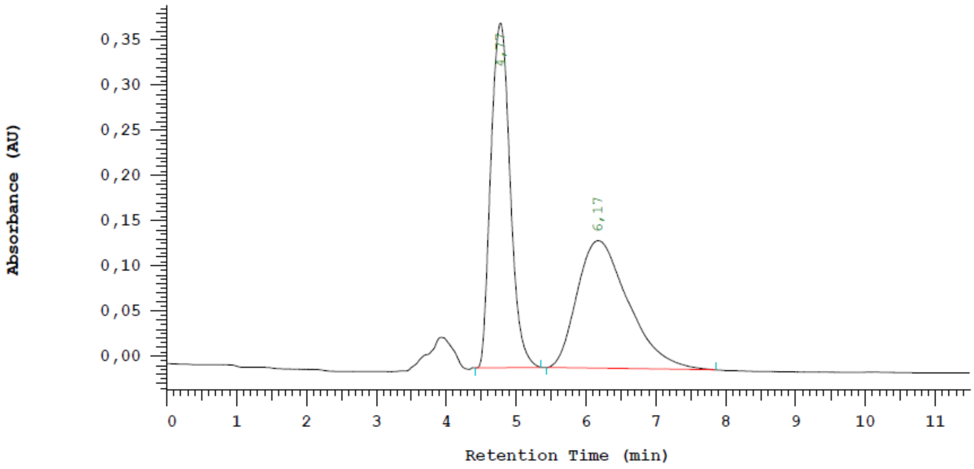

No.	RT	Area	Area %	Name
1	4,77	3655370	50,375	
2	6,17	3600990	49,625	
		7256360	100,000	

8. References

1. Li, C.; Zhang, F.; Shen, Z. *Tetrahedron*, **2020**, *76*, 131727.
2. Winters, G.; Sala, A.; De Paoli, A.; Conti, M. *Synthesis* **1984**, 1050-1052.
